# Identification and Validation of a Potential Stemness-Associated Biomarker in Hepatocellular Carcinoma

**DOI:** 10.1155/2022/1534593

**Published:** 2022-07-11

**Authors:** Yangyang Zhang, Ruike Zhang, Lingxiu Zeng, Haizhou Wang, Ruyi Peng, Meng Zhang, Hailin Zhang, Zhenwei Yang, Liping Gao, Meng Wang, Jing Liu

**Affiliations:** ^1^Department of Gastroenterology, Zhongnan Hospital of Wuhan University, Wuhan, China; ^2^Hubei Clinical Center & Key Lab of Intestinal & Colorectal Diseases, Wuhan University, Wuhan, China

## Abstract

**Background:**

Cancer stem cells (CSCs) are typically related to metastasis, recurrence, and drug resistance in malignant tumors. However, the biomarker and mechanism of CSCs need further exploration. This study is aimed at comprehensively depicting the stemness characteristics and identify a potential stemness-associated biomarker in hepatocellular carcinoma (HCC).

**Methods:**

The data of HCC patients from The Cancer Genome Atlas (TCGA) were collected and divided based on the mRNA expression-based stemness index (mRNAsi) in this study. Weighted gene coexpression network analysis (WGCNA) and the protein-protein interaction (PPI) network were performed, and the genes were screened through the Cytoscape software. Then, we constructed a prognostic expression signature using the multivariable Cox analysis and verified using the GEO and ICGC databases. Even more importantly, we used the three-dimensional (3D) fibrin gel to enrich the tumor-repopulating cells (TRCs) to validate the expression of the signature in CSCs by quantitative RT-PCR.

**Results:**

mRNAsi was significantly elevated in tumor and high-mRNAsi score was associated with poor overall survival in HCC. The positive stemness-associated (blue) module with 737 genes were screened based on WGCNA, and Budding uninhibited by benzimidazoles 1 (BUB1) was identified as the hub gene highly related to stemness in HCC. Then, the prognostic value and stemness characteristics were well validated in the ICGC and GSE14520 cohorts. Further analysis showed the expression of BUB1 was elevated in TRCs.

**Conclusion:**

BUB1, as a potential stemness-associated biomarker, could serve as a therapeutic CSCs-target and predicted the clinical outcomes of patients with HCC.

## 1. Introduction

Liver cancer, the fourth leading cause of cancer-related death, seriously endangers health globally with an estimated incidence of more than 100,000 cases by 2025 [1]. Hepatocellular carcinoma (HCC) is the most common kink of liver cancer, accounting for exceeding 90% of cases. Chronic alcohol consumption, diabetes or obesity-related nonalcoholic steatohepatitis (NASH), and HBV or HCV infection are the primary risk factors for HCC [1]. Over the past decades, some molecular therapies such as sorafenib and immunotherapies have been proven to be efficacy [2]. However, a large proportion of patients were unresponsive to these treatments because of recurrence and metastasis [3].

Cancer stem cells (CSCs), a subgroup of tumor cells that have the ability to self-renew and produce heterogeneous tumor cells, are responsible for cancer metastasis, recurrence, and drug resistance [4, 5]. Recently, mounting evidence suggests that CSCs-target therapy is promising in tumor treatment [6, 7]. Therefore, there is an urgent need to develop new therapies that can effectively inhibit CSCs in HCC. Malta et al. [8] used the one-class logistic regression machine learning algorithm (OCLR) to get the mRNA expression-based stemness index (mRNAsi) and epigenetically regulated-mRNAsi (EREG-mRNAsi) for tumors in the TGCA database. Their research primarily showed that stemness features extracted from transcriptomic data from TCGA tumors could reveal new anticancer therapeutic targets [8]. Many researchers have recently used this index to investigate the characteristics of CSCs in various tumors and the therapeutic targets, such as colorectal cancer [9], lung adenocarcinoma [10], pancreatic ductal adenocarcinoma [11], gastric cancer, and esophagus cancer [12, 13]. The biomarker identified by mRNAsi within these studies mostly related to the impressive progress of tumors and the poor prognosis of patients.

Weighted gene coexpression network analysis (WGCNA) is a biological method to explore genes that are highly correlated with different phenotypes. In this study, we intended to screen out the stemness-associated biomarker using WGCNA in HCC. BUB1 (Budding uninhibited by benzimidazoles 1) was identified as the interested gene highly related to stemness and served as a predictor of prognosis in HCC. As a serine/threonine kinase, BUB1 was described as a core component of the spindle assembly checkpoint (SAC) [14] to prevent errors in chromosome segregation [15, 16]. Although studies have reported the aberrant BUB1 expression was associated with poor survival of HCC patients, no one digs into the connection between the abnormal expression and stemness features in HCC [17–20].

We have used three-diameter (3D) fibrin gel to culture tumor cells in our previous study. We demonstrated that 90 Pa (1 mg/ml) fibrin gel could promote the growth and selection of multicellular colonies of melanoma [21]. These tumor cells have similar characteristics as CSCs and were also called tumor repopulating cells (TRCs) [21]. Then, we also used the 3D fibrin gel to successfully enrich the colon TRCs which were examined by colony formation, tumorigenicity, and drug resistance, and the stemness markers, such as CD133, CD44, SOX2, and OCT4, were also verified to be upregulated [22, 23]. There have been reportsthat CSCs promote the malignant characteristics of HCC and clarified the importance of CSCs in treatment [24]. But, the investigation on the characteristics and potential biomarkers of CSCs in HCC remains lacking. Therefore, our present study identified BUB1 was highly related to mRNAsi through bioinformatic analysis and confirmed the upregulation of BUB1 expression in TRCs through experiments *in vitro*. These results imply that BUB1 could serve as a potential stemness-associated related to prognosis biomarker and CSCs-target therapy strategy for HCC.

## 2. Materials and Methods

### 2.1. Data Collection and Processing

We collected the gene expression, mutation, and clinical details of 374 HCC patients and 50 normal samples from the UCSC Xena website (https://xenabrowser.net/). The mRNAsi index of 368 HCC patients were downloaded from the study published by Malta et al. listed in Table S1 [8]. We divided HCC patients into the high-mRNAsi and low-mRNAsi groups based on the median mRNAsi index (Table S2). The raw gene microarray expression data of GSE14520 and International Cancer Genome Consortium (ICGC-LIRI-JP) and associated clinical information were downloaded from the NCBI Gene Expression Omnibus (GEO) (http://www.ncbi.nlm.nih.gov/geo/) and International Cancer Genome Consortium (ICGC, http://www.icgc.org). Datasets with missing clinical information were excluded. We calculated the average value for those genes corresponding to more than one probe and eliminated the probes matched with more than one gene.

### 2.2. Weighted Gene Coexpression Network Construction

We performed a gene coexpression network using the “WGCNA” R package [25] to analyze and identify gene modules strongly associated with mRNAsi. Genes were sorted based on the median absolute deviation (MAD) value > 1 and the top 5,000 ranked genes were used in the analysis. When the degree of independence was above 0.9 and the average connectivity degree is relatively higher, the appropriate soft threshold power parameter *β* (*β* = 7) was determined [25]. Modules with similar expression proles were merged with a merging threshold of 0.25. The minimum number of genes in each module was set as 30.

### 2.3. Identification of Stemness-Based Module and Hub Gene

The correlation between mRNAsi/EGFR-mRNAsi and module eigengenes (MEs) was used to evaluate module-trait associations. MEs were considered the major component in the principal component analysis for each module. Then, we calculated the correlation between MEs and mRNAsi to identify the relevant module. Gene significance (GS), which was defined by the minus log of a *p* value in the linear regression between gene expression and mRNAsi, was measured to evaluate correlation strength [25]. The positive module highly correlated to mRNAsi was selected as the key module. To explore the potential mechanism of how module genes regulate cancer cell stemness, we uploaded the genes in the key module into the Metascape database [26] and performed the pathway and process enrichment analysis.

Candidate genes were defined by module connectivity, measured by the absolute value of Pearson's correlation (cor. module membership (MM) > 0.8), and clinical trait relationship of mRNAsi, measured by the absolute value of Pearson's correlation (cor. gene significance (GS) > 0.2). Then, we uploaded these genes to the Search Tool for the Retrieval of Interacting Gene (STRING) database (https://string-db.org/) and construct protein-protein interaction (PPI) network [27]. Subsequently, we used the “CytoHubba” and “MCODE” application of Cytoscape software (version 3.8.2) which provided the calculated results by maximum neighborhood component (MNC), degree, bottleneck, stress, radiality, and closeness methods to identify hub gene from the PPI network [28].

### 2.4. Hub Gene Validation

To verify the reliability of the hub gene, GSE14520 and ICGC databases were downloaded. The mRNA differential expression levels of te hub gene between tumor and normal tissues in various cancer types and correlation with TP53 mutation were retrieved from the Tumor Immune Estimation Resource (TIMER 2.0) (http://timer.comp-genomics.org/) [29]. We analyzed the prognostic effect of the hub gene on patients, which was performed using the Sangerbox tools (http://www.sangerbox.com/tool).

### 2.5. GSEA and GSVA of Hub Gene

To further understand the biological function of BUB1 in HCC, GSEA was performed using “ClusterProfiler” R package [30]. The GO and KEGG items were ranked by their enrichment scores, and *p* < 0.05 was considered significant. GSVA [31] is a method to calculate the score of a certain pathway or a signature through the transcriptomic data. All hallmark gene sets were downloaded from the Molecular Signature Database (MSigDB, http://www.gsea-msigdb.org/gsea). Utilizing the “GSVA” and “limma” R package, differential analyses were subsequently conducted based on the score, and the signatures with *p* < 0.05 were defined as significant differentially expressed signatures. The results were visualized by using the “Heatmap” R package.

### 2.6. Multivariate Prognosis Model Construction

The expression of the hub gene and clinical characteristics were combined to be analyzed using multivariate Cox regression analysis to determine whether the hub gene was an independent risk factor in both the TCGA and ICGC databases. We used the “rms” R package to build a prognosis nomogram and draw calibration curves to compare the expected and observed survival probabilities.

### 2.7. Cell Lines and Cell Culture

Human hepatocellular cancer cell lines Hep3B and Huh7 and colon cancer cell lines HT29 and HCT116 were obtained from China Center for Type Culture Collection (CTCC, Wuhan, China). Cells were seeded on a rigid flask with DMEM (HyClone, USA) or RPMI 1640 (HyClone, USA) containing 10% fetal bovine serum (FBS) (Gibico, USA) at 37°C with 5% CO_2_.

### 2.8. Cell Culture in Three-Dimensional (3D) Fibrin Gels

For 3D cell culture, we purchased the salmon fibrinogen and thrombin from Searun Holdings Company (SanDiego, CA, USA). In brief, fibrinogen and cell solution were 1 : 1 mixed to make 1 mg/ml fibrinogen/cell solution, corresponding to 90 Pa in elastic stiffness [21]. Next, 250 *μ*l of fibrinogen/cell mixture and 5 *μ*l of thrombin (100 U/ml; Searun Holdings) were well mixed to coat 24-well plates, which were then incubated for 30 min in a 37°C with 5% CO_2_ incubator. Finally, DMEM or MEM with 10% FBS was added. Tumor spheroids were harvested using Dispase *Π* (Roche, Switzerland) after culturing for 5 days. Tumor cells cultured in the flask were used as control cells. At least three independent experiments were performed for each cell culture experiment.

### 2.9. Quantitative Real-Time PCR

Both of the conventional stemness genes and hub gene expression was further validated at the mRNA level using quantitative RT-PCR (qRT-PCR). Total RNA of cells was extracted using TRIzol reagent according to the supplier's instructions (Invitrogen, USA). Reverse transcription was performed using Transcript First-strand cDNA Synthesis SuperMix (Roche, USA). qRT-PCR was conducted with Ultra SYBR mixture (Cwbio, China) on Roche LightCycler 96 according to standard PCR condition. The primer sequences are provided as follows (human): CD44, CTGCCGCTTTGCAGGTGTA (forward) and CATTGTGGGCAAGGTGC-TATT (reverse); SOX2, TACAGCATGTCCTACTCGCAG (forward) and GAGGAAGAGGTAAC-CACAGGG (antisense); NANOG, CTCCAACATCCTGAACCTCAGC (forward) and CGTCACACCATTGCTATTCTTCG (reverse); and BUB1, GCTCTGTCAGCAGACTTCCTTC (forward) and GCTCTGTCA-GCAGACTTCCTTC (reverse).

### 2.10. Statistical Analysis

Two-tailed Student's *t*-test or ANOVA was used for the significance of differences between groups. Statistical analyses were performed with GraphPad Prism software (v9.0). Other statistical analysis was implemented by R software (v4.0.5). Statistical significance was defined as *p* < 0.05.

## 3. Results

### 3.1. Correlation between mRNAsi and Clinical Characteristics in HCC

**Figure 1:**
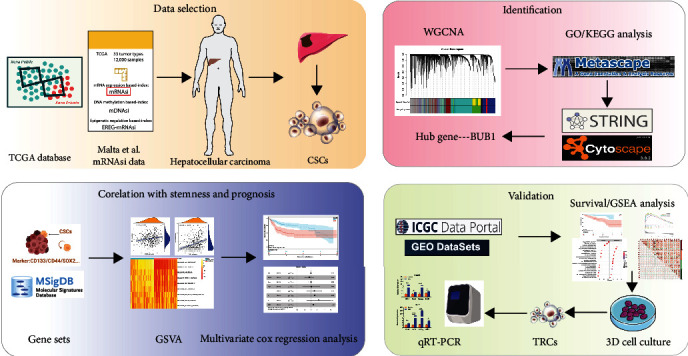
Flow diagram presenting the process of the study.

**Figure 2:**
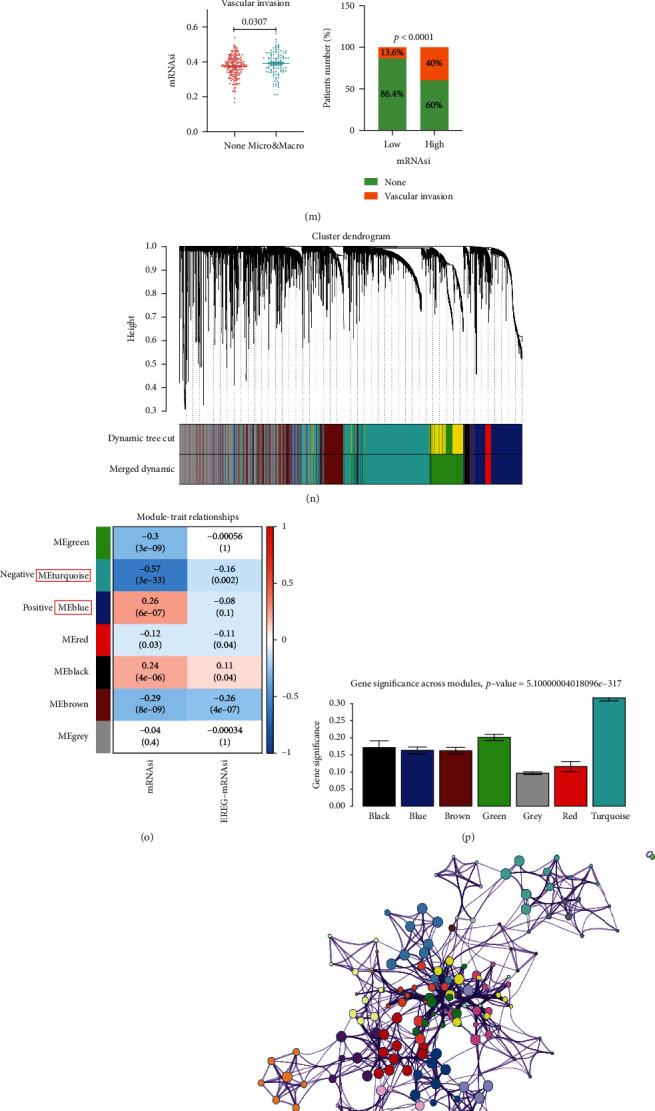
Relationship between the mRNAsi and clinical characteristics and weighted gene coexpression network analysis (WGCNA). (a) Differences in mRNAsi between normal and tumor tissues. (b) Survival curves of high/low mRNAsi group in HCC patients. (c–m) Differences in mRNAsi and clinical characteristics: age (c), gender (d), grade (e), stage (f), fetoprotein value (g), cancer status (h), T/N/M stages (i–k), Ishak score (l), or vascular invasion (m). (n) The cluster dendrogram was based on the expression data of the top 5,000 genes with the MAD value > 1 by WGCNA. (o) Heatmap of correlation between ME and mRNAsi/EGFR-mRNAsi. (p) Distribution of average gene significance and errors in the modules associated with mRNAsi. (q) Scatter plot of MEs in the blue module. (r, s) GO and KEGG enrichment analyses of genes in blue (r) and turquoise (s) module by Metascape.

First of all, the flow diagram was shown to describe this study design (Figure 1). Clinicopathological characteristics of HCC patients were listed in Table S3. As shown, mRNAsi of the tumor was significantly higher than normal tissues, and survival analysis indicated that the high mRNAsi predicted poor prognosis compared with the low mRNAsi subgroup (*p* = 0.0028) (Figures 2(a) and 2(b)). Then, HCC patients were classified by age, gender, TNM stage, tumor stage, fetoprotein value, cancer status, Ishak score, and vascular invasion, respectively; for which, mRNAsi was not significantly associated with age (*p* = 0.9), gender (*p* = 0.063), T (*p* = 0.85), *N* (*p* = 0.42), M (*p* = 0.63), stage (*p* = 0.43), and Ishak score (*p* = 0.32), but significantly associated with tumor grade (*p* = 0.0028), fetoprotein value (*p* = 0.0029), cancer status (*p* = 0.042), and vascular invasion (*p* = 0.0307) (Figure 2(c)–2(l)). Moreover, patients with vascular invasion accounted for 40% in high mRNAsi subgroup while patients with vascular invasion accounted for 13.6% in low mRNAsi subgroup (*p* < 0.0001) (Figure 2(m)).

### 3.2. Construction of Weighted Coexpression Network and Identification of Key Modules

A total of 368 HCC patients with mRNAsi score were included using the “WGCNA” R package [25]. The thresholding power of *β* = 7 (scale-free *R*^2^ = 0.94) (Figure S1A-C) was selected to construct a scale-free network (Figure 2(n)) and 7 modules were identified. Of these modules, the turquoise module had the highest negative correlation with mRNAsi (*r* = −0.57, *p* = 3*e* − 33) and the blue module had the highest positive correlation with mRNAsi (*r* = 0.26, *p* = 6*e* − 7) (Figures 2(o)–2(q); Figure S1D). To reveal the biological role of these two modular genes in biological processes, GO and KEGG enrichment analyses were performed in the Metascape tool. Herein, genes in the blue module were primarily enriched in “mitotic cell cycle,” “G2/M checkpoints,” “PID PLK1 PATHWAY,” “DNA conformation change” (Figure 2(r)), and genes in the turquoise module were enriched for “NABA CORE MATRISOME,” “external encapsulating structure organization,” “extracellular matrix organization,” and “blood vessel development” (Figure 2(s)). Since we wanted to find a stemness-associated biomarker, the blue module containing 737 genes, which was positively related to mRNAsi (Table S4), was selected for further analysis.

### 3.3. Identification the Hub Gene

**Figure 3:**
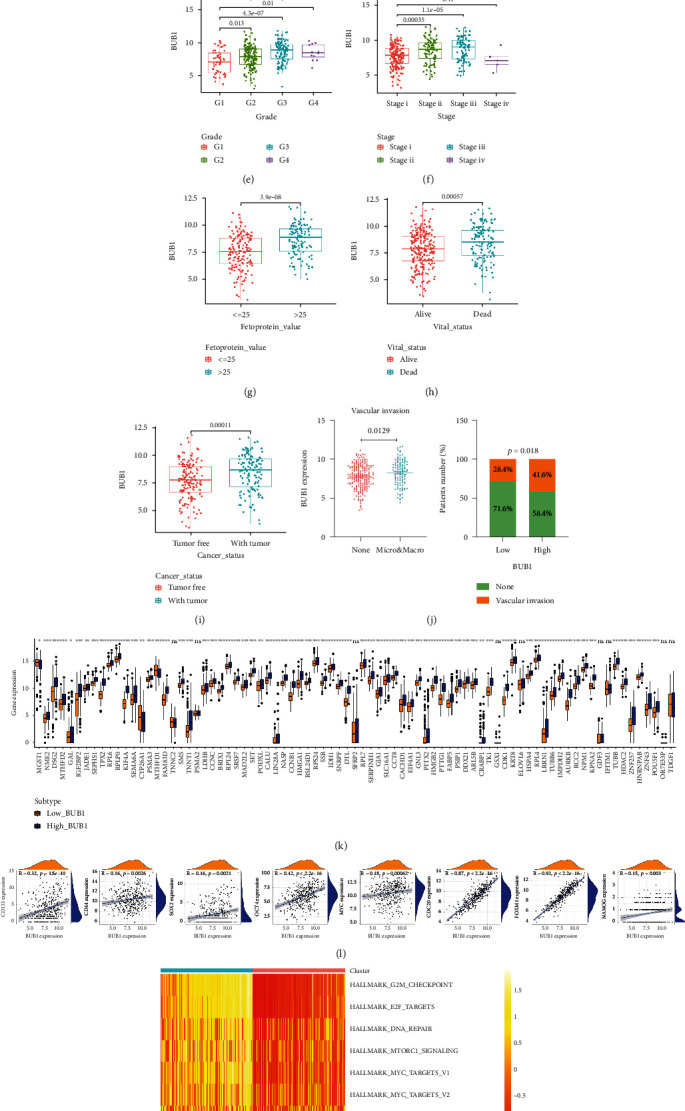
Protein-protein network (PPI) construction and the relationship between hub gene BUB1, clinical, and stemness characteristics in HCC samples from the TCGA cohort. (a) The PPI construction of candidate genes in the blue module. (b) The hub gene was identified using the Cytoscape software. (c) The levels of BUB1 in various tumor and normal tissues were examined using TIMER2.0. (d) Correlation between BUB1 expression and mRNAsi group. (e–k) Differences in BUB1 expression and clinical characteristics: grade (e), stage (f), fetoprotein value (g), vital status (h), cancer status (i), or vascular invasion (j). (k) The correlations between BUB1 expression and upregulated genes in human embryonic stem cell lines from MSigDB. (l) The correlations between BUB1 expression and specific stemness markers. (m, n) Heatmap of the significantly differential hallmarks and pathways of BUB1 subtypes in HCC.

Under the threshold of the MM higher than 0.8 and the GS higher than 0.2, 112 candidate genes were chosen to perform the PPI network in the STRING database (minimum required interaction score: 0.4) (Table S5). These consisted of 111 nodes and 4342 edges (Figure 3(a)). Through using 9 topological analysis methods from the CytoHubba to sort the PPI network nodes, we found that the Budding uninhibited by benzimidazoles 1 (BUB1) score ranks in the top 10 of the 9 algorithms (Figure 3(b), Table 1). Furthermore, by performing gene module analysis using the MCODE, BUB1 was also found in cluster 1 (MCODE score = 63.40) (Table S6). Therefore, BUB1 was selected as the hub gene for further validation. In addition, BUB1 mRNA levels were significantly increased in most types of tumor than normal tissues in the TIMER 2.0 database (Figure 3(c)). In the TCGA dataset, the BUB1 expression level was significantly upregulated in high mRNAsi compared with the low mRNAsi subgroup (*p* < 0.001) (Figure 3(d)).

### 3.4. Correlation between BUB1 Expression and Clinical Characteristics in HCC

Similarly, we analyzed the correlation between the BUB1 expression and clinical features. Patients older than 60 years of age do not show a significant increase in BUB1 expression (*p* = 0.082), as well as the result of correlation with gender, child, N, and M stage (*p* > 0.05) (Figure S2A-F). Compared with grade1, stage i, and T stage, the expression of BUB1 showed a higher trend in grade 2/3/4, stage ii/iii, and T 2/3/4, but the difference was also not significant (*p* > 0.05) (Figures 3(e) and 3(f); Figure S2D). It was worth noting that compared with fetoprotein value ≤ 25 *μ*g/l, the status of alive and tumor-free showed higher levels of BUB1 in patients with fetoprotein value > 25 *μ*g/l, the status of death, and with tumor (*p* < 0.001) (Figures 3(g)–3(i)). Then, patients were divided into high and low subtypes according to the median expression of BUB1. We found the BUB1 expression was significantly associated with vascular invasion (*p* = 0.0129), and patients with vascular invasion accounted for 41.6% in high BUB1 subtype while patients with vascular invasion accounted for 28.4% in low BUB1 subtype (*p* = 0.018) (Figure 3(j)). We visualized the mutational features utilizing the “maftools” R package in HCC. In summary, we found the missense mutation accounts for the majority of the mutation classifications, the single-nucleotide variants (SNP) occurring the most frequently, and C >A was the top type of SNP class (Figure S3A). Besides, we exhibited the top mutated genes, including TP53 (Figure S3B). Therefore, we explored and discovered the BUB1 expression was significantly higher in the TP53-mutant group than in the TP53-WT group in HCC using the TIMER2.0 database (*p* < 0.001) (Figure S3C).

### 3.5. Stemness Characteristics of BUB1 Subtypes

First, we downloaded the upregulated genes list in six human embryonic stem cell lines tested from MSigDB (Table S7). As shown in Figure 3(k), BUB1 expression was positively correlated with most of the genes in the list. Previous studies had shown that cancer stem cells maintain their stem cell-like biological characteristics through a high expression of specific stemness markers (such as SOX2, CD44, CD133, and MYC). Therefore, we identified that there was a significant positive correlation with the expression of CD133, CD44, SOX2, OCT4, CDC20, FOXM1, NANOG, and MYC (*p* < 0.001) (Figure 3(l)). Subsequently, GSVA was performed to analyze potential biological characteristics of BUB1 in HCC patients. According to HALLMARK gene sets defined by MSigDB, high BUB1 subtype was significantly enriched in “G2/M checkpoint,” “DNA repair,” “cell cycle,” “DNA replication,” and “WNT/MYC/NORCH/HEDGEHOG/mTOR signaling pathway,” which were established hallmarks and pathways associated with cancer cell proliferation and stemness (Figures 3(m) and 3(n)).

### 3.6. Prognostic Value of BUB1 in HCC Patients

**Figure 4:**
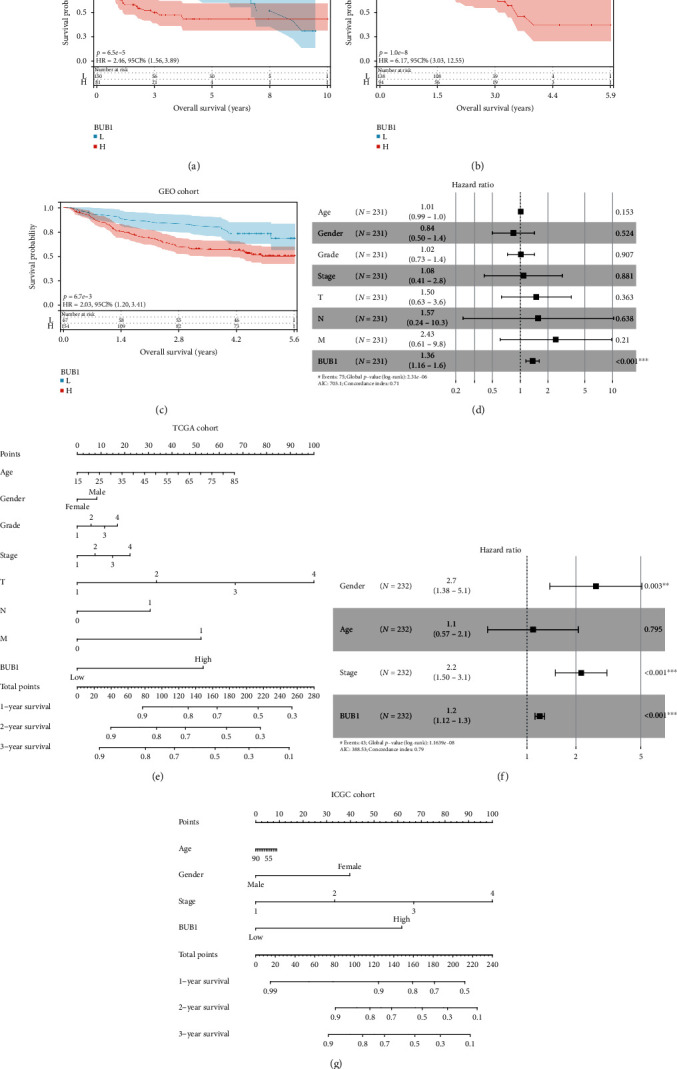
The prognosis value of BUB1 in HCC patients from the TCGA, GEO, and ICGC cohorts. (a–c) Survival analysis of BUB1 subtypes in HCC from the TGGA (a), ICGC (b), and GSE14520 (c) cohorts. (d, f) Multivariate Cox regression analysis of clinical features and BUB1 expression in TCGA (d) and ICGC (f) cohorts. (e, g) Prognostic nomogram on the basis of clinical features and BUB1 expression in the TCGA (e) and ICGC (g) cohorts.

We next investigate the prognostic value of BUB1 in HCC. Survival analysis showed a significant difference between high and low BUB1 subtypes in the TCGA, ICGC, and GSE14520 cohorts (*p* < 0.001) (Figures 4(a)–4(c)). Then, multivariate Cox regression analysis was performed among the clinical variables. After controlling for other confounding factors, BUB1 expression remained an independent predictor of overall survival (OS) in both the TCGA (HR = 1.36, 95%CI = 1.16–1.6, *p* < 0.001) (Figure 4(d)) and the ICGC cohorts (HR = 1.2, 95%CI = 1.12–1.3, *p* < 0.001) (Figure 4(f)). Meanwhile, we established a nomogram that could better predict the survival of HCC patients and visualized the prediction results, which showed that the nomogram composed of BUB1 expression and clinical phenotype was effective both in the TCGA and ICGC cohorts (Figures 4(e) and 4(g)). The calibration curve also demonstrated good capacity for the nomogram between prediction and observation in both of the databases (Figure S4A, B). These results indicated that the BUB1 expression could predict the prognosis of HCC patients independently.

### 3.7. Validation of Stemness Characteristics of BUB1 in External Database

**Figure 5:**
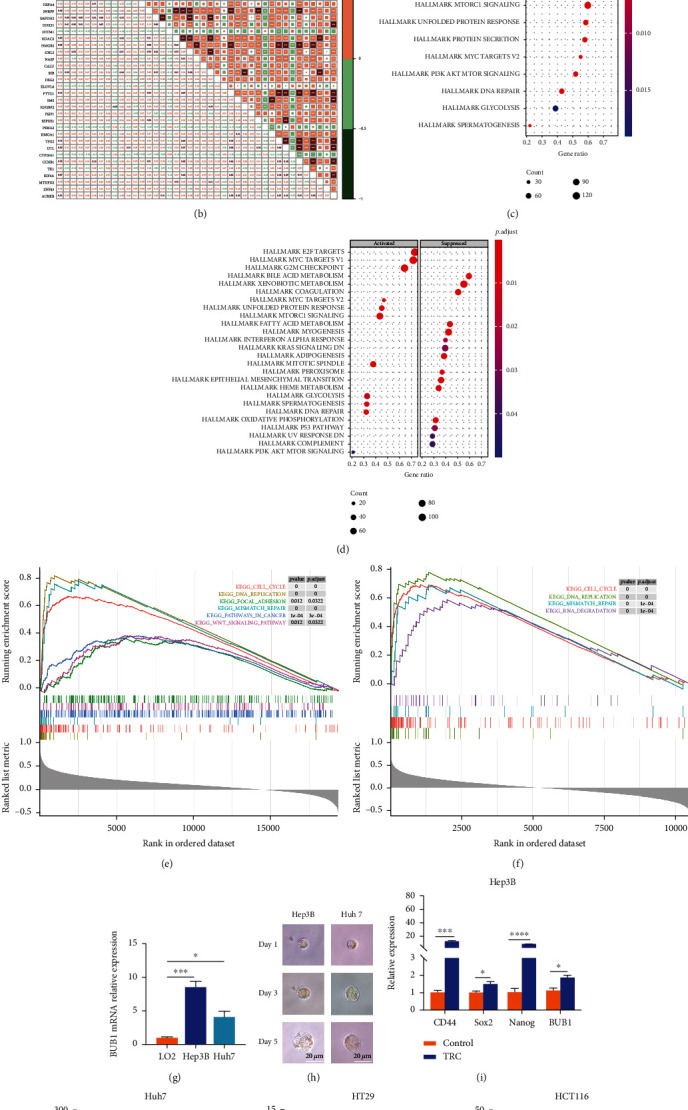
Validation of stemness characteristics and expression of BUB1 in HCC patients from the GEO and ICGC cohorts and TRCs. (a, b) The correlations between BUB1 expression and upregulated genes in human embryonic stem cell lines in the ICGC (a) and GSE14520 (b) cohorts. (c–f) GSEA of BUB1 in the ICGC (c, e) and GSE14520 (d, f) cohorts. (g) qRT-PCR of BUB1 expression in Hep3B and Huh7 vs. LO2. (h) Representative images of liver TRC spheroids. Scale bars, 20 *μ*m. (i, j) qRT-PCR of BUB1 expression in Hep3B and Huh7 liver TRCs vs. control cells. (k, l) qRT-PCR of BUB1 expression in HT29 and HCT116 colon TRCs vs. control cells. Data are shown as the mean ± SEM (*n* = 3); ^∗^*p* < 0.05, ^∗∗^*p* < 0.01, ^∗∗∗^*p* < 0.001, and ^∗∗∗∗^*p* < 0.0001.

The stemness-associated features of BUB1 were further validated in the ICGC and GSE14520 cohorts. BUB1 expression was significantly positively correlated with stemness-related genes in the ICGC cohort (Figure 5(a)) and the GSE14520 cohort (Figure 5(b)). KEGG and GO enrichment analyses also confirmed that the genes in the high BUB1 subtype were enriched in the cell cycle and related stemness pathways both in the ICGC cohort (Figures 5(c) and 5(e)) and the GSE14520 cohort (Figures 5(d) and 5(f)). Then, we verified the BUB1expression was significantly elevated in HCC cell lines than normal liver cell line (Figure 5(g)). To further validate the BUB1 expression in HCC cancer stem cells, we explored the mRNA expression patterns of BUB1 in TRCs of HCC which had the same characteristics as the CSCs using established TRCs 3D enrichment methods (Figure 5(h)). It was significantly upregulated in TRCs of both Hep3B and Huh7 than control cells (*p* < 0.05), which was consistent with the validation results of the database (Figures 5(i) and 5(j)). Interestingly, we got the same results in TRCs of colorectal cancer cells (Figures 5(k) and 5(l)).

## 4. Discussion

HCC, as the main type of liver cancer, is an ongoing challenge for public health. Therapies to prevent and treat recurrence and metastasis of HCC are still inadequate and ineffective. CSCs have self-renewal capacities that drive tumorigenesis and aberrant differentiation, and CSCs are responsible for cellular heterogeneity, recurrence, metastasis, and therapy resistance [32]. To gain insights into the stemness characteristics of HCC, WGCNA was conducted to explore the modules related to mRNAsi, followed by hub gene selection and survival analysis (Figure 1). Our results showed the potential value of BUB1 as a biomarker of stemness and survival for HCC.

As well known, levels of AFP, tumor grade, or vascular invasion are the prognostic indictors of HCC [1, 33–35]. Among them, vascular invasion is a primary factor of tumor recurrence and metastasis. A study was using the markers that reflect aggressive tumor characteristics, such as vascularization, P53 overexpression, and biliary/stem cell markers, and found it seems adequate to the reality with effects in OS, feasible in biopsies, which may identify patients that could benefit from aggressive treatments [34]. So, we initially analyzed the relationship between clinical features and mRNAsi in HCC and found the HCC tissues exhibited a higher mRNAsi as the pathological tumor grade elevated and with the vascular invasion, and the higher mRNAsi predicted the shorter OS, which was in accordance with the research in ovarian cancer, colorectal cancer, and esophageal cancer [9, 12, 36].

Then, we constructed a coexpression network using WGCNA to compute the key module highly related to mRNAsi in HCC. Results demonstrated that the turquoise and blue modules took the highest negative and positive weights. GO and KEGG showed that genes in the blue module were particularly enriched in the regulation of the cell cycle. These results suggest that genes in the blue module may play a role in improving the self-renewal and proliferative abilities of CSCs, and genes or proteins involved in regulating the cell cycle may be a target for anti-CSC therapies. Furthermore, when combined with a PPI network and Cytoscape software, BUB1 was identified as the hub gene and might serve as a potential stemness-associated biomarker and correlated with poor prognosis in HCC.

BUB1 is a serine/threonine kinase that prevents errors in chromosome segregation in mitosis [15, 16]. As the central component of the mitotic checkpoint for SAC, BUB1 is essential for chromosome congression and kinetochore location [14, 15]. Moreover, several studies have shown that inhibiting spindle checkpoints may be a viable cancer treatment method. Previous studies have shown that the BUB1 mutation causes a high rate of chromosomal *mis*-segregation, accompanied by growth defects and premature senescence [16]. These studies disclosed that BUB1 plays a vital role in changing cell progression, including the CSCs. However, studies on the role of BUB1 in the stemness characteristics of CSCs in HCC remain poorly understood. To further explore the role of BUB1 in cancer progression, we performed a pan-cancer study from the TCGA cohorts using TIMER2.0 and unveiled that BUB1 was upregulated in numerous cancers. In vitro, we found a higher BUB1 expression level in HCC cell lines than normal liver cell lines. The high BUB1 subtype had a higher mRNAsi value than the low BUB1 subtype, implying that the high BUB1 subtype had more inherent heterogeneity and a protumorigenic role than the low BUB1 subtype. It has been reported that depleting BUB1 could reduce cancer stem cell potential in a breast cancer cell line, resulting in inhibiting the formation of xenografts in mice [37]. Similarly, high BUB1 expression with higher mRNAsi score was significantly associated with the aggressive progression of HCC, such as tumor grade and vascular invasion, which reflected that the BUB1 was contributed to the progression of HCC. Additionally, the high BUB1 expression subtype predicted shorter survival than the low expression subtype, consistent with the study of ovarian cancer [36], gastric cancer [38], and pancreatic ductal adenocarcinoma [11]. These results suggested the high BUB1 expression associated with high stemness may partly contribute to the poor outcome of HCC. Recent studies have tried to establish morphological and immunohistochemical patterns that could be important to individualized treatments to assess a better classification for the clinical reality [34, 39]. In Tsujikawa and his colleagues research, they found the biliary/stem cell marker-positive group exhibited more aggressive features, such as poor tumor differentiation and increased frequency of portal vein invasion, and also the group has the shortest time to recurrence among the three groups [39]. So, immunohistochemical profiling could reflect tumor aggressiveness. BUB1 might serve as a potential biomarker in the biopsy samples for morphophenotypic analysis and the overall survival correlation of HCC. And it could be more feasible in biopsies with routine antibodies for identifying patients with aggressive HCC, which was more beneficial in less developed institutions.

The association between BUB1 and stemness may be related to various mechanisms. In glioblastoma (GBM), BUB1 was highly expressed [40, 41] with elevated expression related to poor prognosis and radioresistance in GBM patients. Mechanistically, BUB1 was directly regulated by FOXM1, a transcription factor [41]. According to GSVA analysis, the genes in the high BUB1 subtype were clustered mostly in the hallmark of G2M checkpoint, E2F targets, and DNA repair, suggesting that BUB1 plays an important role in maintaining cell growth and viability and may affect cancer stemness features through regulating the cell cycle. The research found BUB1 could promote the formation of the mitotic checkpoint complex (MCC) and phosphorylate CDC20, which are necessary for spindle point signaling. The inactivation of any one of these mechanisms will cause checkpoint defects, leading to chromosomal *mis*-segregation and aneuploidy, which are associated with cancer [42]. Furthermore, the BUB1-BUB3 complex worked with telomeric repeat binding factor 1 (TRF1) and promoted the recruitment of BLM helicase to maintain and promote telomere replication [43]. In addition, transforming growth factor-*β* (TGF-*β*) signal transduction, which regulates cell proliferation and differentiation, may be regulated by BUB1 [44]. In osteosarcoma, inhibition of BUB1 markedly suppressed cell proliferation, cell migration, and invasion through blocking of the PI3K/Akt and ERK signaling pathways [45]. Our results confirmed that BUB1 plays a vital role in the regulation of MYC, Wnt, Notch, and Hedgehog pathways, which are sharply associated with stemness signaling [6, 8]. This may indicate a probability of BUB1 in the regulation mechanism of CSCs in HCC. Thus, a reasonable conclusion can be drawn that BUB1 can not only regulate the cell cycle of CSCs but also influence the cellular stemness-related pathway to promote cancer progression and enhance the stemness capacity. In other words, BUB1 may play a role in enhancing the self-renewal and proliferation properties of CSCs.

CSCs can be isolated through specific biomarkers on the cell surface, such as CD133, CD44, and SOX2 [6]. The correlation analysis pointed out that BUB1 was significantly positive correlated with the classic stemness biomarkers (i.e., CD44, SOX2, and NANOG). Human embryonic stem (huES) cells have the ability to differentiate into a variety of cell lines, *Bhattacharya B* and his colleagues used high-quality microarray to identify and provide a distinct set of the “stemness” signature which was upregulated in 6 human embryonic stem cell lines [46]. We downloaded these genes and explored their correlation with BUB1, and we got a similar result. These findings indicate that samples with high stemness biomarker expression levels may have a high BUB1 expression level. Or, the BUB1 expression level was higher in CSCs than in normal cancer cells. Interestingly, with the help of GEVA, we found the KEGG of the P53 signaling pathway was significantly enriching the high BUB1 subtype. As we all know, TP53 is the most common mutation in HCC. Mutations of TP53 can not only lose their tumor-suppressive functions but also promote tumorigenesis as well as promote the self-renewal and differentiation of CSCs [47]. Moreover, it has been found that driving cells into premature senescence by P53 is dependent on BUB1 binding [48, 49]. Therefore, we explored that a higher BUB1 expression level in the TP53-mutant group than in the WT-TP53 group, which implied that cells overexpressed BUB1 to promote tumorigenesis may have a connection with TP53 mutation in HCC.

More importantly, we used 3D fibrin gel to enrich liver TRCs, which had similar characteristics as CSCs and verified that the BUB1 expression was higher than control cells, as same as in colon TRCs. Simultaneously, the biomarkers of CSCs, such as CD44 and SOX2, were upregulated in TRCs. We have to mention some defects in our study. First, the prognostic effect of BUB1 needs to be validated in more samples and a prospective cohort of patients containing follow-up data. Then, more basic research is required to systematically elucidate the underlying stemness molecular mechanisms of BUB1 *in vitro* and *in vivo*.

Taken together, using the coexpression analysis, BUB1 was identified as a potential stemness-associated biomarker related to prognosis in HCC. The high expression of BUB1 in HCC might be one of the reasons that CSCs maintain stemness characteristics. Therefore, we provide evidence that BUB1 might serve as a new therapeutic CSCs-target in HCC, such as the BUB1-specific inhibitors, and contribute to better understanding the CSCs-related molecular mechanism of the metastasis and recurrence of HCC. However, the conclusion we draw from bioinformatics analysis and limited experiments needs more basic research to verify.

## Figures and Tables

**Table 1:** Top genes were shown using CytoHubba in Cytoscape software.

Category	Rank methods in CytoHubba					
	Bottleneck	Stress	Radiality	MNC	Degree	Closeness
1	BUB1	TTK	CDCA8	CDCA8	CDCA8	CDCA8
2	ORC1	CDCA8	CDK1	CDK1	CDK1	CDK1
3	UHRF1	BUB1	BUB1	BUB1	BUB1	BUB1
4	ASF1B	CDK1	TTK	CCNA2	TTK	TTK
5	TTK	CCNA2	CCNA2	KIF20A	CCNA2	CCNA2
6	RAD51	KIF20A	KIF20A	BUB1B	KIF20A	KIF20A
7	KIF2C	BUB1B	BUB1B	TTK	BUB1B	BUB1B
8	PBK	CCNB1	CCNB1	CCNB1	CCNB1	CCNB1
9	PRC1	TOP2A	AURKB	AURKB	AURKB	AURKB
10	ASPM	AURKB	TOP2A	TOP2A	TOP2A	TOP2A

## Data Availability

The datasets supporting the conclusions of this article were available in the TCGA database (https://portal.gdc.cancer.gov), ICGC database (http://www.icgc.org), and GEO database (http://www.ncbi.nlm.nih.gov/geo). The raw data supporting the conclusions of this article accompanies are included in the article and supplementary material. Further inquiries can be directed to the corresponding author.

## Supplementary Materials

Supplementary Materials Figure S1: determination of soft-thresholding power in WGCNA. (A) Analysis of the scale-free fit index and the mean connectivity for various soft-threshold powers (*β* = 7). (B) Histogram of connectivity distribution when *β* = 7. (C) Checking the scale free topology when *β* = 7. (D) Scatter plot of MEs in the turquoise module. Figure S2: differences in BUB1 expression and clinical characteristics in HCC: age (A), gender (B), child (C), T stage (D), M stage (E), and N stage (F). Figure S3: the mutational features in HCC. (A) The landscape of mutation in HCC. (B) The top 30 mutation genes in HCC. (C) The correlation between TP53 mutation and BUB1 expression using TIMER2.0. Supplementary Figure S4: the calibration curve for the nomogram in TCGA database (A) and ICGC database (B). Table S1: the mRNAsi score of HCC patients in the TCGA database. Table S2: the mRNAsi subtype of HCC patients in the TCGA database. Table S3: clinicopathological characteristics of HCC patients from the TCGA, ICGC, and GEO cohorts. Table S4: 737 genes in blue module by WGCNA. Table S5: 112 genes involved in PPI (MM > 0.8; GS > 0.2). Table S6: the result of MCODE method in Cytoscape. Table S7: The ‘stemness' signature in MSigDB: genes upregulated and common to 6 human embryonic stem cell lines tested.
