# Supplementary material for: Identification and Validation of a Potential Stemness-Associated Biomarker in Hepatocellular Carcinoma
Source: Stem Cells Int. 2022 Jul 11;2022:1534593. doi: 10.1155/2022/1534593 (PMC9293570; doi:10.1155/2022/1534593)
Supplement: Supplementary Materials — Figure S1: determination of soft-thresholding power in WGCNA. (A) Analysis of the scale-free fit index and the mean connectivity for various soft-threshold powers (β = 7). (B) Histogram of connectivity distribution when β = 7. (C) Checking the scale free topology when β = 7. (D) Scatter plot of MEs in the turquoise module. Figure S2: differences in BUB1 expression and clinical characteristics in HCC: age (A), gender (B), child (C), T stage (D), M stage (E), and N stage (F). Figure S3: the mutational features in HCC. (A) The landscape of mutation in HCC. (B) The top 30 mutation genes in HCC. (C) The correlation between TP53 mutation and BUB1 expression using TIMER2.0. Supplementary Figure S4: the calibration curve for the nomogram in TCGA database (A) and ICGC database (B). Table S1: the mRNAsi score of HCC patients in the TCGA database. Table S2: the mRNAsi subtype of HCC patients in the TCGA database. Table S3: clinicopathological characteristics of HCC patients from the TCGA, ICGC, and GEO cohorts. Table S4: 737 genes in blue module by WGCNA. Table S5: 112 genes involved in PPI (MM > 0.8; GS > 0.2). Table S6: the result of MCODE method in Cytoscape. Table S7: The ‘stemness' signature in MSigDB: genes upregulated and common to 6 human embryonic stem cell lines tested. [file 1534593.f1.zip › Table S1.pdf]

Table S1. The mRNAsi score of HCC patients in TCGA database.

ID	mRNAsi	EREG-mRNAsi
TCGA.2V.A95S.01A	0.402031387	0.6270183
TCGA.2Y.A9GS.01A	0.44229679	0.6181242
TCGA.2Y.A9GT.01A	0.300767799	0.515315
TCGA.2Y.A9GU.01A	0.37526763	0.4058798
TCGA.2Y.A9GV.01A	0.310258058	0.5519246
TCGA.2Y.A9GW.01A	0.325594706	0.6265033
TCGA.2Y.A9GX.01A	0.256914765	0.5855435
TCGA.2Y.A9GY.01A	0.467310563	0.7211716
TCGA.2Y.A9GZ.01A	0.398377177	0.6308551
TCGA.2Y.A9H0.01A	0.512638883	0.5212234
TCGA.2Y.A9H1.01A	0.428315371	0.6380647
TCGA.2Y.A9H2.01A	0.373167971	0.4853749
TCGA.2Y.A9H3.01A	0.343728269	0.4747591
TCGA.2Y.A9H4.01A	0.444588211	0.7182007
TCGA.2Y.A9H5.01A	0.302689004	0.589496
TCGA.2Y.A9H6.01A	0.272552931	0.4731827
TCGA.2Y.A9H7.01A	0.415275223	0.5275674
TCGA.2Y.A9H8.01A	0.434944669	0.4750669
TCGA.2Y.A9H9.01A	0.390972704	0.4971089
TCGA.2Y.A9HA.01A	0.410491047	0.5850479
TCGA.2Y.A9HB.01A	0.378925631	0.525902
TCGA.3K.AAZ8.01A	0.421625445	0.6889969
TCGA.4R.AA8I.01A	0.394800838	0.4816988
TCGA.5C.A9VG.01A	0.42189577	0.6075373
TCGA.5C.A9VH.01A	0.301258148	0.5661142
TCGA.5C.AAPD.01A	0.420491015	0.6870335
TCGA.5R.AA1C.01A	0.422825083	0.6930022
TCGA.5R.AA1D.01A	0.214527172	0.721845
TCGA.5R.AAAM.01A	0.273470066	0.7313958
TCGA.BC.4072.01B	0.337306104	0.7053867
TCGA.BC.4073.01B	0.378525437	0.7119936
TCGA.BC.A10Q.01A	0.301563782	0.4278185
TCGA.BC.A10R.01A	0.288976455	0.4925389
TCGA.BC.A10S.01A	0.30972238	0.5948244
TCGA.BC.A10T.01A	0.359388181	0.7443341
TCGA.BC.A10U.01A	0.381205381	0.5792695
TCGA.BC.A10W.01A	0.45921262	0.6416361
TCGA.BC.A10X.01A	0.27907277	0.565045
TCGA.BC.A10Y.01A	0.462289616	0.6107905
TCGA.BC.A10Z.01A	0.46646496	0.5961774
TCGA.BC.A110.01A	0.249282971	0.6204828
TCGA.BC.A112.01A	0.401500344	0.6114923
TCGA.BC.A216.01A	0.417796017	0.5478731
TCGA.BC.A217.01A	0.472463511	0.7081109
TCGA.BC.A3KF.01A	0.402669323	0.5297258
TCGA.BC.A3KG.01A	0.454465593	0.5910141

TCGA.BC.A5W4.01A	0.441268809	0.6565135
TCGA.BC.A69H.01A	0.462115638	0.8174163
TCGA.BC.A69I.01A	0.320756265	0.6415133
TCGA.BC.A8YO.01A	0.450591479	0.536462
TCGA.BD.A2L6.01A	0.4222915	0.6058226
TCGA.BD.A3EP.01A	0.292602758	0.6101177
TCGA.BW.A5NO.01A	0.426322699	0.652451
TCGA.BW.A5NP.01A	0.423895173	0.6064979
TCGA.BW.A5NQ.01A	0.538778668	0.7049025
TCGA.CC.5258.01A	0.444544107	0.6835728
TCGA.CC.5259.01A	0.418498753	0.6450539
TCGA.CC.5260.01A	0.305091563	0.5574592
TCGA.CC.5261.01A	0.323826036	0.6161455
TCGA.CC.5262.01A	0.355584388	0.7492907
TCGA.CC.5263.01A	0.412725318	0.5921995
TCGA.CC.5264.01A	0.455407636	0.5427846
TCGA.CC.A123.01A	0.457048608	0.7189743
TCGA.CC.A1HT.01A	0.398298264	0.6981167
TCGA.CC.A3M9.01A	0.391949986	0.5864358
TCGA.CC.A3MA.01A	0.325286264	0.73494
TCGA.CC.A3MB.01A	0.408020796	0.6075951
TCGA.CC.A3MC.01A	0.405648671	0.6258257
TCGA.CC.A5UC.01A	0.426954481	0.6934122
TCGA.CC.A5UD.01A	0.359148816	0.7769623
TCGA.CC.A5UE.01A	0.468794533	0.64143
TCGA.CC.A7IE.01A	0.417866953	0.5686571
TCGA.CC.A7IF.01A	0.400887722	0.5830157
TCGA.CC.A7IG.01A	0.422419314	0.6688389
TCGA.CC.A7IH.01A	0.330009293	0.6752528
TCGA.CC.A7II.01A	0.520585976	0.6980796
TCGA.CC.A7IJ.01A	0.343349	0.5819262
TCGA.CC.A7IK.01A	0.428169128	0.5937163
TCGA.CC.A7IL.01A	0.461128786	0.7862397
TCGA.CC.A8HS.01A	0.415075711	0.5034755
TCGA.CC.A8HT.01A	0.497121163	0.6664876
TCGA.CC.A8HU.01A	0.452671909	0.4793852
TCGA.CC.A8HV.01A	0.393709008	0.6067752
TCGA.CC.A9FS.01A	0.422264306	0.5072979
TCGA.CC.A9FU.01A	0.473129993	0.6025308
TCGA.CC.A9FV.01A	0.195650393	0.6289958
TCGA.CC.A9FW.01A	0.381859416	0.5764036
TCGA.DD.A113.01A	0.405738975	0.5572528
TCGA.DD.A114.01A	0.361393826	0.5068985
TCGA.DD.A115.01A	0.376120625	0.4887181
TCGA.DD.A116.01A	0.384494474	0.6435401
TCGA.DD.A118.01A	0.374641228	0.5155321
TCGA.DD.A119.01A	0.393059698	0.6089842
TCGA.DD.A11A.01A	0.426664522	0.5287009

TCGA.DD.A11B.01A	0.380866606	0.5456799
TCGA.DD.A11C.01A	0.386526604	0.549391
TCGA.DD.A11D.01A	0.328945871	0.6010198
TCGA.DD.A1EA.01A	0.418243542	0.659767
TCGA.DD.A1EB.01A	0.37346333	0.5736277
TCGA.DD.A1EC.01A	0.380408465	0.5402993
TCGA.DD.A1ED.01A	0.276780286	0.5595666
TCGA.DD.A1EE.01A	0.409259746	0.6166049
TCGA.DD.A1EF.01A	0.374939783	0.4789254
TCGA.DD.A1EG.01A	0.400450907	0.6911694
TCGA.DD.A1EH.01A	0.36532964	0.529474
TCGA.DD.A1EI.01A	0.310474787	0.6186306
TCGA.DD.A1EJ.01A	0.421237345	0.6028696
TCGA.DD.A1EK.01A	0.359057612	0.5867842
TCGA.DD.A1EL.01A	0.45898646	0.8080613
TCGA.DD.A39V.01A	0.412734927	0.6922034
TCGA.DD.A39W.01A	0.361751369	0.6708133
TCGA.DD.A39X.01A	0.420783488	0.6828031
TCGA.DD.A39Y.01A	0.491215697	0.678218
TCGA.DD.A39Z.01A	0.435935597	0.6085345
TCGA.DD.A3A1.01A	0.452212205	0.6754447
TCGA.DD.A3A2.01A	0.378894549	0.5479468
TCGA.DD.A3A3.01A	0.442435043	0.6842149
TCGA.DD.A3A4.01A	0.427227531	0.7337799
TCGA.DD.A3A5.01A	0.406669684	0.7152836
TCGA.DD.A3A6.01A	0.211989092	0.5367421
TCGA.DD.A3A7.01A	0.451230645	0.5708721
TCGA.DD.A3A8.01A	0.354039239	0.6432146
TCGA.DD.A3A9.01A	0.316245661	0.5598708
TCGA.DD.A4NA.01A	0.300081654	0.5181366
TCGA.DD.A4NB.01A	0.191298434	0.6183703
TCGA.DD.A4ND.01A	0.22975882	0.504921
TCGA.DD.A4NE.01A	0.389009516	0.5246918
TCGA.DD.A4NF.01A	0.326269311	0.5698548
TCGA.DD.A4NG.01A	0.359498268	0.6428035
TCGA.DD.A4NH.01A	0.343796561	0.5601602
TCGA.DD.A4NI.01A	0.304640072	0.5756534
TCGA.DD.A4NJ.01A	0.389383225	0.5140851
TCGA.DD.A4NK.01A	0.366395045	0.5367031
TCGA.DD.A4NL.01A	0.276101628	0.6070019
TCGA.DD.A4NN.01A	0.380719639	0.5826845
TCGA.DD.A4NO.01A	0.341850228	0.5902436
TCGA.DD.A4NP.01A	0.328091441	0.5928132
TCGA.DD.A4NQ.01A	0.425754415	0.6360607
TCGA.DD.A4NR.01A	0.40302257	0.6624471
TCGA.DD.A4NS.01A	0.167069581	0.5365024
TCGA.DD.A4NV.01A	0.291853992	0.5935596
TCGA.DD.A73A.01A	0.387148757	0.646046

TCGA.DD.A73B.01A	0.464942752	0.5539556
TCGA.DD.A73C.01A	0.354957334	0.5046693
TCGA.DD.A73D.01A	0.46390155	0.6870656
TCGA.DD.A73E.01A	0.350252768	0.5953816
TCGA.DD.A73F.01A	0.429181289	0.6407559
TCGA.DD.A73G.01A	0.417477561	0.6261116
TCGA.DD.AA3A.01A	0.377334731	0.4208524
TCGA.DD.AAC8.01A	0.369215218	0.6849967
TCGA.DD.AAC9.01A	0.336896346	0.7191775
TCGA.DD.AACA.01A	0.366490783	0.679449
TCGA.DD.AACB.01A	0.416731896	0.7240275
TCGA.DD.AACC.01A	0.381311145	0.6730823
TCGA.DD.AACD.01A	0.43447763	0.6768644
TCGA.DD.AACE.01A	0.369165049	0.5589356
TCGA.DD.AACF.01A	0.459436425	0.5406837
TCGA.DD.AACG.01A	0.526861399	0.7025845
TCGA.DD.AACH.01A	0.392067191	0.5176557
TCGA.DD.AACI.01A	0.399266162	0.5968356
TCGA.DD.AACJ.01A	0.455668992	0.639425
TCGA.DD.AACK.01A	0.413389067	0.6653049
TCGA.DD.AACL.01A	0.478640804	0.6334648
TCGA.DD.AACN.01A	0.292954013	0.5680099
TCGA.DD.AACO.01A	0.438940164	0.5672531
TCGA.DD.AACP.01A	0.475996959	0.6637976
TCGA.DD.AACQ.01A	0.407147672	0.4749463
TCGA.DD.AACS.01A	0.423782906	0.5554683
TCGA.DD.AACT.01A	0.346282769	0.722242
TCGA.DD.AACU.01A	0.358327248	0.667004
TCGA.DD.AACV.01A	0.496065375	0.5395985
TCGA.DD.AACW.01A	0.439320813	0.7078076
TCGA.DD.AACX.01A	0.438482767	0.6015167
TCGA.DD.AACY.01A	0.375078605	0.5606947
TCGA.DD.AACZ.01A	0.456028815	0.66803
TCGA.DD.AAD0.01A	0.41558194	0.6302246
TCGA.DD.AAD1.01A	0.252927096	0.5993607
TCGA.DD.AAD2.01A	0.317213083	0.6396936
TCGA.DD.AAD3.01A	0.271081519	0.6349571
TCGA.DD.AAD5.01A	0.415938821	0.7088233
TCGA.DD.AAD6.01A	0.48611698	0.5261061
TCGA.DD.AAD8.01A	0.410427912	0.5414179
TCGA.DD.AADA.01A	0.309994627	0.5375884
TCGA.DD.AADB.01A	0.435940228	0.6659571
TCGA.DD.AADC.01A	0.441300902	0.6985257
TCGA.DD.AADD.01A	0.482728088	0.5956693
TCGA.DD.AADF.01A	0.480083897	0.5578276
TCGA.DD.AADG.01A	0.370284914	0.7094744
TCGA.DD.AADI.01A	0.351242839	0.5950415
TCGA.DD.AADJ.01A	0.390220053	0.648143

TCGA.DD.AADK.01A	0.341677466	0.5882902
TCGA.DD.AADL.01A	0.420094241	0.6273474
TCGA.DD.AADM.01A	0.443701183	0.7236908
TCGA.DD.AADN.01A	0.564322993	0.7646809
TCGA.DD.AADO.01A	0.47116611	0.7124268
TCGA.DD.AADP.01A	0.366347798	0.4696046
TCGA.DD.AADQ.01A	0.445425878	0.6153075
TCGA.DD.AADR.01A	0.495596604	0.7024195
TCGA.DD.AADS.01A	0.438865734	0.6763277
TCGA.DD.AADU.01A	0.380491143	0.6394853
TCGA.DD.AADV.01A	0.38310278	0.5389126
TCGA.DD.AADW.01A	0.340946814	0.4823017
TCGA.DD.AADY.01A	0.397050583	0.5577807
TCGA.DD.AAE0.01A	0.392650824	0.6220177
TCGA.DD.AAE1.01A	0.412693337	0.5842323
TCGA.DD.AAE2.01A	0.387491538	0.4962874
TCGA.DD.AAE3.01A	0.354168558	0.7340942
TCGA.DD.AAE4.01A	0.441971883	0.709927
TCGA.DD.AAE6.01A	0.514440989	0.5143474
TCGA.DD.AAE7.01A	0.310360147	0.688587
TCGA.DD.AAE9.01A	0.397604103	0.6424538
TCGA.DD.AAEA.01A	0.440954402	0.5911455
TCGA.DD.AAEB.01A	0.367846079	0.6457544
TCGA.DD.AAED.01A	0.420746769	0.5253835
TCGA.DD.AAEE.01A	0.400954119	0.5825011
TCGA.DD.AAEG.01A	0.399662386	0.6563217
TCGA.DD.AAEH.01A	0.393716179	0.5954165
TCGA.DD.AAEI.01A	0.463445545	0.6577051
TCGA.DD.AAEK.01A	0.325496775	0.5985998
TCGA.DD.AAVP.01A	0.374294476	0.5808224
TCGA.DD.AAVQ.01A	0.376290822	0.6675618
TCGA.DD.AAVR.01A	0.320553409	0.6153605
TCGA.DD.AAVS.01A	0.375798816	0.6970518
TCGA.DD.AAVU.01A	0.488229727	0.6810627
TCGA.DD.AAVV.01A	0.328084959	0.6467004
TCGA.DD.AAVW.01A	0.272509041	0.5897505
TCGA.DD.AAVX.01A	0.383138242	0.657738
TCGA.DD.AAVY.01A	0.421611602	0.6699441
TCGA.DD.AAVZ.01A	0.404742976	0.6505097
TCGA.DD.AAW0.01A	0.323632201	0.6427564
TCGA.DD.AAW1.01A	0.348154144	0.5784483
TCGA.DD.AAW2.01A	0.379845672	0.6158622
TCGA.DD.AAW3.01A	0.394061288	0.6046867
TCGA.ED.A459.01A	0.420212678	0.5500208
TCGA.ED.A4XI.01A	0.267618652	0.5891717
TCGA.ED.A5KG.01A	0.322490991	0.5702782
TCGA.ED.A627.01A	0.20823974	0.5600396
TCGA.ED.A66X.01A	0.344243717	0.6748066

TCGA.ED.A66Y.01A	0.356780008	0.4611744
TCGA.ED.A7PX.01A	0.3480764	0.675495
TCGA.ED.A7PY.01A	0.385130964	0.6002011
TCGA.ED.A7PZ.01A	0.438983413	0.6534058
TCGA.ED.A7XO.01A	0.377698271	0.5398758
TCGA.ED.A7XP.01A	0.352233507	0.621656
TCGA.ED.A82E.01A	0.289304277	0.6310721
TCGA.ED.A8O5.01A	0.364398553	0.5141574
TCGA.ED.A8O6.01A	0.403046831	0.636233
TCGA.ED.A97K.01A	0.260007756	0.6344966
TCGA.EP.A12J.01A	0.378368901	0.597646
TCGA.EP.A26S.01A	0.377926304	0.6622521
TCGA.EP.A2KA.01A	0.406398211	0.7298248
TCGA.EP.A2KB.01A	0.38200201	0.5693096
TCGA.EP.A2KC.01A	0.352516456	0.6204743
TCGA.EP.A3JL.01A	0.395250219	0.6394185
TCGA.EP.A3RK.01A	0.417863733	0.6877218
TCGA.ES.A2HS.01A	0.370900958	0.5730798
TCGA.ES.A2HT.01A	0.38717631	0.6969944
TCGA.FV.A23B.01A	0.373810832	0.5441806
TCGA.FV.A2QQ.01A	0.40145493	0.6741314
TCGA.FV.A2QR.01A	0.322481292	0.6715117
TCGA.FV.A3I0.01A	0.254598306	0.3690733
TCGA.FV.A3I1.01A	0.37148863	0.6119071
TCGA.FV.A3R2.01A	0.434427591	0.6503047
TCGA.FV.A3R3.01A	0.228858993	0.6473625
TCGA.FV.A495.01A	0.38086704	0.5933076
TCGA.FV.A496.01A	0.375096533	0.5191962
TCGA.FV.A4ZP.01A	0.480008573	0.8262163
TCGA.FV.A4ZQ.01A	0.46372555	0.6660887
TCGA.G3.A25S.01A	0.497042761	0.6202899
TCGA.G3.A25T.01A	0.334060683	0.4504548
TCGA.G3.A25U.01A	0.4671218	0.7081591
TCGA.G3.A25V.01A	0.306916374	0.5753412
TCGA.G3.A25X.01A	0.368099641	0.6462773
TCGA.G3.A25Y.01A	0.336551226	0.701083
TCGA.G3.A25Z.01A	0.394260316	0.6992946
TCGA.G3.A3CH.01A	0.372589388	0.6282046
TCGA.G3.A3CI.01A	0.31684894	0.6819198
TCGA.G3.A3CJ.01A	0.397076959	0.7018962
TCGA.G3.A3CK.01A	0.390696029	0.6167013
TCGA.G3.A5SI.01A	0.465125197	0.5089257
TCGA.G3.A5SJ.01A	0.339752124	0.5700464
TCGA.G3.A5SK.01A	0.337120006	0.4282319
TCGA.G3.A5SL.01A	0.363978336	0.711655
TCGA.G3.A5SM.01A	0.341561119	0.5589423
TCGA.G3.A6UC.01A	0.398493053	0.6518087
TCGA.G3.A7M5.01A	0.445357284	0.6343956

TCGA.G3.A7M6.01A	0.270556147	0.4926731
TCGA.G3.A7M7.01A	0.387498554	0.697945
TCGA.G3.A7M8.01A	0.337296988	0.6095244
TCGA.G3.A7M9.01A	0.465847273	0.598513
TCGA.G3.AAUZ.01A	0.412786107	0.650547
TCGA.G3.AAV0.01A	0.374232871	0.4695443
TCGA.G3.AAV1.01A	0.389031761	0.6412009
TCGA.G3.AAV2.01A	0.343080466	0.539451
TCGA.G3.AAV3.01A	0.357436651	0.5964427
TCGA.G3.AAV4.01A	0.392326253	0.6460427
TCGA.G3.AAV5.01A	0.456684681	0.7152864
TCGA.G3.AAV6.01A	0.402072532	0.6285859
TCGA.G3.AAV7.01A	0.409434255	0.6364029
TCGA.GJ.A3OU.01A	0.292243941	0.6600596
TCGA.GJ.A6C0.01A	0.366663842	0.5573975
TCGA.GJ.A9DB.01A	0.329617519	0.5686128
TCGA.HP.A5MZ.01A	0.270255574	0.6037752
TCGA.HP.A5N0.01A	0.276216557	0.6133456
TCGA.K7.A5RF.01A	0.278350975	0.6384758
TCGA.K7.A5RG.01A	0.400378942	0.4970109
TCGA.K7.A6G5.01A	0.35687489	0.6831146
TCGA.K7.AAU7.01A	0.262504471	0.4770926
TCGA.KR.A7K0.01A	0.299155846	0.4888581
TCGA.KR.A7K2.01A	0.33519873	0.4859627
TCGA.KR.A7K7.01A	0.480736215	0.6274042
TCGA.KR.A7K8.01A	0.356514765	0.6781085
TCGA.LG.A6GG.01A	0.374384152	0.5542329
TCGA.LG.A9QC.01A	0.349269982	0.7185978
TCGA.LG.A9QD.01A	0.352649055	0.5692721
TCGA.MI.A75C.01A	0.452561506	0.6655109
TCGA.MI.A75E.01A	0.341760363	0.6844671
TCGA.MI.A75G.01A	0.397396459	0.5678613
TCGA.MI.A75H.01A	0.378783541	0.6728029
TCGA.MI.A75I.01A	0.425210142	0.613705
TCGA.MR.A520.01A	0.360867091	0.6654485
TCGA.MR.A8JO.01A	0.233304077	0.5624606
TCGA.NI.A4U2.01A	0.347909823	0.5436514
TCGA.NI.A8LF.01A	0.280371166	0.6289511
TCGA.O8.A75V.01A	0.361047226	0.6493412
TCGA.PD.A5DF.01A	0.330034824	0.6580602
TCGA.QA.A7B7.01A	0.490004345	0.5926191
TCGA.RC.A6M3.01A	0.482410159	0.6735867
TCGA.RC.A6M4.01A	0.426913936	0.6463146
TCGA.RC.A6M5.01A	0.240609072	0.5987204
TCGA.RC.A6M6.01A	0.420895875	0.9478249
TCGA.RC.A7S9.01A	0.43852242	0.3938508
TCGA.RC.A7SB.01A	0.383446766	0.7393782
TCGA.RC.A7SF.01A	0.363763802	0.5081269

TCGA.RC.A7SH.01A	0.407888066	0.5111912
TCGA.RC.A7SK.01A	0.390480219	0.7869953
TCGA.RG.A7D4.01A	0.494866752	0.6141403
TCGA.UB.A7MA.01A	0.343807827	0.6281047
TCGA.UB.A7MB.01A	0.451732963	0.5508755
TCGA.UB.A7MC.01A	0.432233362	0.5011966
TCGA.UB.A7MD.01A	0.338971176	0.6303257
TCGA.UB.A7ME.01A	0.369710049	0.5563016
TCGA.UB.A7MF.01A	0.433116143	0.4719438
TCGA.UB.AA0U.01A	0.342683157	0.5412653
TCGA.UB.AA0V.01A	0.268808511	0.5525878
TCGA.WJ.A86L.01A	0.384842502	0.5679349
TCGA.WQ.A9G7.01A	0.433125085	0.4889908
TCGA.WQ.AB4B.01A	0.364400792	0.6805573
TCGA.WX.AA44.01A	0.34577802	0.5210923
TCGA.WX.AA46.01A	0.3312014	0.5194283
TCGA.WX.AA47.01A	0.423841758	0.4772703
TCGA.XR.A8TC.01A	0.411732188	0.6714204
TCGA.XR.A8TD.01A	0.340036774	0.7481323
TCGA.XR.A8TE.01A	0.275569579	0.6639444
TCGA.XR.A8TF.01A	0.44778585	0.5597489
TCGA.XR.A8TG.01A	0.337954481	0.5402404
TCGA.YA.A8S7.01A	0.305093689	0.6119264
TCGA.ZP.A9CV.01A	0.369955393	0.5348249
TCGA.ZP.A9CY.01A	0.306328798	0.5628491
TCGA.ZP.A9CZ.01A	0.43114296	0.5510082
TCGA.ZP.A9D0.01A	0.372313002	0.4387602
TCGA.ZP.A9D1.01A	0.315447381	0.6527536
TCGA.ZP.A9D2.01A	0.325387294	0.5681305
TCGA.ZP.A9D4.01A	0.396879413	0.5564343
TCGA.ZS.A9CD.01A	0.326130795	0.6223145
TCGA.ZS.A9CE.01A	0.42123616	0.7046985
TCGA.ZS.A9CF.01A	0.394743071	0.5888328
TCGA.ZS.A9CG.01A	0.301896012	0.7299359
