# Supplementary material for: Identification and Validation of a Potential Stemness-Associated Biomarker in Hepatocellular Carcinoma
Source: Stem Cells Int. 2022 Jul 11;2022:1534593. doi: 10.1155/2022/1534593 (PMC9293570; doi:10.1155/2022/1534593)
Supplement: Supplementary Materials — Figure S1: determination of soft-thresholding power in WGCNA. (A) Analysis of the scale-free fit index and the mean connectivity for various soft-threshold powers (β = 7). (B) Histogram of connectivity distribution when β = 7. (C) Checking the scale free topology when β = 7. (D) Scatter plot of MEs in the turquoise module. Figure S2: differences in BUB1 expression and clinical characteristics in HCC: age (A), gender (B), child (C), T stage (D), M stage (E), and N stage (F). Figure S3: the mutational features in HCC. (A) The landscape of mutation in HCC. (B) The top 30 mutation genes in HCC. (C) The correlation between TP53 mutation and BUB1 expression using TIMER2.0. Supplementary Figure S4: the calibration curve for the nomogram in TCGA database (A) and ICGC database (B). Table S1: the mRNAsi score of HCC patients in the TCGA database. Table S2: the mRNAsi subtype of HCC patients in the TCGA database. Table S3: clinicopathological characteristics of HCC patients from the TCGA, ICGC, and GEO cohorts. Table S4: 737 genes in blue module by WGCNA. Table S5: 112 genes involved in PPI (MM > 0.8; GS > 0.2). Table S6: the result of MCODE method in Cytoscape. Table S7: The ‘stemness' signature in MSigDB: genes upregulated and common to 6 human embryonic stem cell lines tested. [file 1534593.f1.zip › Table S4.pdf]

Table S4. 737 genes in blue module by WGCNA.
PEG10
TRIM71
PITX1
CYP3A4
IGF2BP1
BEX2
SLCO4C1
UPK3A
NPSR1-AS1
MSI1
GLRB
POF1B
RP11-476K15.1
ZIC5
TTC36
ILDR1
C6orf223
IGF2BP3
COCH
NRCAM
MEP1A
GUCY1B2
KCNK9
NCAPD2P1
LINC00992
GPC5
FOXN4
ELOVL7
SFN
PRR15L
C1orf106
CYP8B1
SUSD4
LINC01224
FOXQ1
TAT
CTSE
EPO
B4GALNT2
PRAME
GCNT3
CCDC162P
KIAA1244
EPPK1
CTSV
GAL3ST1
C1orf186
SULT1C2
LRCOL1
PAQR5
GPC3
SYT8
PYCR1
SLC29A4
BCAS1
ADH4
SLC2A5
TERT
C1QL1
PNCK

SLC5A12
PCLO
FER1L6
LINC01234
RP11-118B18.2
SALL4
LINC01152
GLYAT
BMP8B
HPD
DMBT1
TRAM1L1
RP11-313J2.1
SLC10A1
CDCA7
GLDN
FMO1
LINC00511
FXYD3
VIL1
LYPD1
FOXJ1
FOLR1
STMND1
CYP19A1
UGT8
CD300LG
SALL2
CD24
ADH1C
MAEL
RP11-244M2.1
SGCE
CLGN
LHFPL3-AS2
SMPDL3B
RP5-1120P11.1
C15orf48
GYLTL1B
EVPL
PACSIN1
COL28A1
KB-1460A1.1
RP11-57A1.1
LYPD6
NEB
RP11-108M9.3
RP11-7M8.2
PTPN20A
ZNF239
RP11-875O11.3
NSUN7
TFAP2A-AS1
ANXA13
PCK1
RP4-763G1.2
SPHK1
HERC2P3
ZIC2
TCL6
LINC00944

RP11-187E13.1
SMC1B
SLC22A15
IGF2BP2
TRNP1
CFHR4
C6orf183
C3orf14
CYP26B1
PKIB
EGLN3
IGSF1
GSDMC
MYBL2
MMP1
DUXAP10
STOX1
FER1L4
ABCA8
SIX4
MYO1A
DQX1
CDH8
CCDC155
SPATA17
PPP1R14D
ZPLD1
BFSP2
C12orf56
FAM183A
ERC2
HIST3H2BB
ZNF695
MCCD1
XXyac-YM21GA2.7
CKMT1A
RP11-350E12.4
ERVMER34-1
RP11-567G11.1
CECR7
TCAM1P
HOXA11-AS
RP11-742B18.1
RP11-618I10.2
RP11-501C14.7
RP11-497G19.1
TRAV30
CTD-2555C10.3
RP11-352D13.6
RP1-118J21.25
RP11-353N14.2
RP11-38M8.1
IBSP
LINC01587
PHEX
EYA1
DLX4
HAVCR1
SLC7A10
MYEF2
MIOX

FAM64A
FIBCD1
FIRRE
HMGA2
EFHC2
PDIA2
DCHS2
RP11-132N15.1
AC093375.1
RP11-73M7.1
RP1-45C12.1
RP11-227H15.5
RP11-829H16.3
CYP4F23P
TMEM145
PPM1E
AFP
SOBP
TMEM246
TMED3
TRIM31
RP11-422N16.3
FABP4
LAMA5-AS1
CD109
ASNS
ESYT3
LINC00238
C5orf30
MCOLN3
SULT1C2P1
RIPPLY3
LEFTY1
ALG1L
ADH1B
OTX1
HAGLR
ISL2
KSR2
CLDN18
SPTSSB
RP11-449J21.5
TMEM100
NRSN2
CNKSR1
ZNF883
FUT2
ATOH8
RP11-356M20.1
RNF157
ADAM22
SLC27A5
NEURL3
ATP6V0D2
MFI2
SLITRK5
RP11-528A4.2
LINC01296
LRRC37A6P
GNAZ
MKRN2OS

AQP9
DUSP9
RP11-16E12.2
MCF2L2
B3GNT5
ZNF711
RIBC2
TMIE
C12orf75
NAP1L4P1
TFAP2A
BEND3P1
VASH2
DRP2
PLCH1
ANKRD18CP
TAC3
FAM87A
RP11-452D2.2
RP11-616K6.1
RP11-496I2.5
AC241377.2
DAGLA
INPP5J
KIF18B
SLC38A1
RTKN2
NUP62CL
ZYG11A
KCNN1
APOBEC3B
LINC01485
TBC1D30
CTC-260E6.4
E2F7
GPX7
EIF5A2
RP11-498P14.5
IGSF3
FADS2
PRR36
TICRR
RP11-424C20.2
CTD-2510F5.4
AL589743.1
TTLL7
FHAD1
TP73
SPATC1L
TOP2A
ETV1
DEPDC1B
DDR1
MDK
PLBD1-AS1
ABCC4
ZNF300
COL9A2
IQCD
NANOS1
PLK1

KCNJ11
UHRF1
CLSPN
BUB1B
CXXC4
GTSE1
DNAJC6
MNS1
KIF15
AMH
TNNT1
ELOVL3
DNAI1
ACPT
PLEKHS1
KIF5A
DNAH3
MMP10
CASKIN1
RNF186
C1QL4
LA16c-60H5.7
PSMD10P2
SLC9A7P1
AC118754.4
AL133493.2
RP11-564D11.3
RP11-459D22.1
LL22NC03-N14H11.1
RP11-165A20.3
LINC00189
HP
ZNF93
CALCR
KCNC1
MCM10
BAIAP2L2
RP11-351J23.1
DEPDC1
CDC20
ACOT11
HPR
HOMER1
E2F8
COLCA2
TTK
RP11-381N20.1
RP11-439C15.4
RP11-146E13.5
RAD54L
ITPKA
PIK3CD-AS2
KIF23
KIF20A
UGT1A7
POLQ
PRR19
FOXM1
CENPF
ZSWIM5
SLC1A5

SLC6A9
LINC00654
CDKN2A
ADAMTS6
FBXO41
SH2D5
RP11-34P13.16
KB-1742H10.3
DMC1
LRRC1
HKDC1
TROAP
AURKB
DFNA5
BIRC5
CDCA2
BCL2L14
KCTD17
PFN2
SHOX2
LRRC66
RP11-89H19.2
NEIL3
SKA1
KIF2C
AGPAT4
KIF18A
CEP55
ANLN
CELSR3
CCNE1
CENPA
GINS1
AKR7A3
NEK10
AC015849.16
ADM2
PHGDH
SRSF12
TLDC2
STRIP2
MPP2
SBK1
XRCC2
GFRA3
CNGB3
SIX2
TTLL6
PIFO
ACBD7
GRIN1
CDHR4
LINC00943
MALRD1
CCNI2
CDKN2A-AS1
AC007461.2
SLC25A5P5
RP11-165F24.3
HSPA8P8
RP11-49O14.2

RP11-132N15.2
PTPRG-AS1
CTC-458A3.1
RP11-120K18.2
RP11-973F15.2
RP11-336K24.12
RIMBP3
HIST1H2BF
CTAGE4
LINC00479
CPHL1P
RP11-417L19.2
MKI67
SHCBP1
NUF2
ZC3H12B
ROBO1
BUB1
PIF1
DNASE1L3
CCNB2
FAM72C
RNFT2
KPNA7
TOB2P1
CDT1
NEURL1
CKAP2L
ESM1
ADORA2B
UBE2C
MIR210HG
HCG16
RNU6-850P
DNM1
STK26
LCAT
ZNF738
CTD-2561J22.5
NCAPG
ZNF296
DLGAP5
OR2B6
REG4
FAM57B
LRRFIP1P1
PRR11
ESCO2
PLXNC1
PBK
CENPM
COLCA1
KIFC1
CDK1
BLM
CENPK
SGOL1
MELK
TET1
ERCC6L
KIAA1524

KIAA1324
DTL
RP1-152L7.5
ZC2HC1A
CYP2W1
KIAA1377
PEBP4
FAM227A
ZNF385C
FAM72B
HMSD
RP11-79N23.1
RP11-496H1.1
CTC-378H22.2
RP11-963H4.3
CSPG5
VEGFB
KIF4A
SLC35G2
NCAPH
CAPN12
HJURP
ECT2
TPX2
SNHG4
CENPE
E2F1
CTB-147N14.6
CHAF1B
CIB2
SPOCD1
TRIP13
SAPCD2
GPRIN1
ASPM
USP51
KREMEN2
TRIM36
RP11-398C13.2
ATP2A1-AS1
HELLS
E2F2
CDC6
CDCA8
ESPL1
ASPDH
RP11-923I11.6
RDM1
GSG2
RNF5P1
CDC45
CDC25A
DUSP15
BCYRN1
ENTPD2
MPP3
GABRQ
ASF1B
KIF14
CMB9-22P13.1
DAND5

TEX19
AC010136.2
RP11-495P10.10
ZNF878
CTD-2529O21.1
RP11-353N14.4
RP11-382D12.2
TSSK5P
FIGNL2
DHDH
GRIN3B
REEP2
SCAMP5
SRC
ZNF233
RTN2
PKMYT1
HMMR
RP1-8B1.4
DIAPH3
PLCB1
RSPH14
C7orf31
SLC2A1
SLC44A4
SKA3
NEK2
CDCA7L
KCP
ANKS6
C16orf59
FADS1
TNFRSF11A
FBXO43
MTFR2
FAM81A
DPY19L2P2
FAM225A
LINC01091
CCDC64
CRHR1-IT1
BMF
ARID3A
ENPP2
CCNA2
BEST4
CDKN2B-AS1
RP11-351J23.2
MAFG-AS1
SH2D6
RP11-1008C21.2
FIGN
CENPI
ASRGL1
AC009005.2
FAM46B
SAMD13
KIF12
RP11-486A14.1
WBP5
DSG2

ZNF724P
C6
MCM2
PRC1
CDCA5
SCG5
LINC01268
ORC1
GALNT15
ARNTL2
CTD-2589H19.6
MESP2
LRP12
ARHGAP11A
KIF11
FAM182B
SAPCD1
QPCT
MYB
ORC6
EME1
HIST1H3B
RAD51
PRTFDC1
PLEKHG6
NGFRAP1
CYP27B1
RAD51AP1
EXO1
PAQR4
RHOF
RNF39
TTC34
NPAS1
PTTG1
MPP7
TIGD3
FAM72D
ROCK1P1
C3orf67
TRPC1
LYPD3
RP5-858L17.1
RP11-839D17.3
KIAA0101
PLP2
RMI2
FAM111B
MLK4
GINS4
RPSAP58
CHRNA5
SPC25
G6PD
CCNB1
LMNB1
WDR62
LINC00299
NDC80
OSBP2
NT5DC2

ENPP6
RNASE2
CDKN3
KIF7
ZWINT
TYMS
IQGAP3
RP11-863K10.7
FANCD2
ZNF192P1
AIFM3
RP11-158L12.4
FOXD2
CTC-471F3.5
KLHL7-AS1
FOXD2-AS1
KRBA1
TMC7
ARHGAP11B
STK31
LHX4
CDCA3
FZD6
MAGEE1
GPR19
HIST2H2BF
ARHGEF19
APOC4
RP11-20I20.4
RP11-98G7.1
BNIP3P11
STIL
BRIP1
RP1-193H18.2
MSH5
WDR76
TRIM67
TCEAL8
KITLG
ATP8B2
ASB9
KNTC1
OIP5
AGBL2
MFI2-AS1
ZNF594
GAS2L3
TRIM59
SMC4
KCNMB3
SQLE
RRM2
BCAS4
AP001469.9
MAP2K6
PAFAH1B3
ALDOC
PARPBP
CNTNAP1
PRR7
CDC25C

CENPW
CIT
CCDC78
TCF19
CBX2
MTL5
