# Supplementary material for: Identification and Validation of a Potential Stemness-Associated Biomarker in Hepatocellular Carcinoma
Source: Stem Cells Int. 2022 Jul 11;2022:1534593. doi: 10.1155/2022/1534593 (PMC9293570; doi:10.1155/2022/1534593)
Supplement: Supplementary Materials — Figure S1: determination of soft-thresholding power in WGCNA. (A) Analysis of the scale-free fit index and the mean connectivity for various soft-threshold powers (β = 7). (B) Histogram of connectivity distribution when β = 7. (C) Checking the scale free topology when β = 7. (D) Scatter plot of MEs in the turquoise module. Figure S2: differences in BUB1 expression and clinical characteristics in HCC: age (A), gender (B), child (C), T stage (D), M stage (E), and N stage (F). Figure S3: the mutational features in HCC. (A) The landscape of mutation in HCC. (B) The top 30 mutation genes in HCC. (C) The correlation between TP53 mutation and BUB1 expression using TIMER2.0. Supplementary Figure S4: the calibration curve for the nomogram in TCGA database (A) and ICGC database (B). Table S1: the mRNAsi score of HCC patients in the TCGA database. Table S2: the mRNAsi subtype of HCC patients in the TCGA database. Table S3: clinicopathological characteristics of HCC patients from the TCGA, ICGC, and GEO cohorts. Table S4: 737 genes in blue module by WGCNA. Table S5: 112 genes involved in PPI (MM > 0.8; GS > 0.2). Table S6: the result of MCODE method in Cytoscape. Table S7: The ‘stemness' signature in MSigDB: genes upregulated and common to 6 human embryonic stem cell lines tested. [file 1534593.f1.zip › Table S5.pdf]

Table S5. 112 genes involved in PPI (MM>0.8;GS>0.2)

| probes | moduleColo | GS.mRNAs | MMblue |
|---|---|---|---|
| BIRC5 | blue | 0.5480794 | 0.8394893 |
| CDC20 | blue | 0.5183242 | 0.8585693 |
| CDT1 | blue | 0.5078634 | 0.8145862 |
| CDCA3 | blue | 0.5000184 | 0.854155 |
| CENPM | blue | 0.4965139 | 0.8145303 |
| CDCA5 | blue | 0.4957087 | 0.8764555 |
| AURKB | blue | 0.4952912 | 0.8247067 |
| SKA1 | blue | 0.4932935 | 0.8542025 |
| KIF2C | blue | 0.4863578 | 0.8988066 |
| CCNB1 | blue | 0.4830228 | 0.8770345 |
| KIAA0101 | blue | 0.4821823 | 0.8316257 |
| CDKN3 | blue | 0.477883 | 0.8116518 |
| SPC25 | blue | 0.4727587 | 0.8672233 |
| TROAP | blue | 0.4724648 | 0.87374 |
| CDC25A | blue | 0.4722976 | 0.834748 |
| RRM2 | blue | 0.4712341 | 0.8407224 |
| SGOL1 | blue | 0.4682513 | 0.8741676 |
| SKA3 | blue | 0.4671768 | 0.8615604 |
| KIFC1 | blue | 0.4654692 | 0.880652 |
| NCAPG | blue | 0.4645724 | 0.8887634 |
| EXO1 | blue | 0.4643559 | 0.8715783 |
| MYBL2 | blue | 0.4629405 | 0.8873049 |
| NDC80 | blue | 0.4602471 | 0.8722108 |
| CENPA | blue | 0.4569154 | 0.8887041 |
| CDC45 | blue | 0.4523283 | 0.85788 |
| EME1 | blue | 0.4515794 | 0.8662482 |
| ZWINT | blue | 0.4507242 | 0.8650088 |
| UBE2C | blue | 0.4474032 | 0.8371126 |
| CCNB2 | blue | 0.446908 | 0.8796941 |
| NUF2 | blue | 0.4451898 | 0.8854652 |
| OIP5 | blue | 0.4450036 | 0.8367603 |
| HJURP | blue | 0.4439909 | 0.8920543 |
| KIF4A | blue | 0.4436071 | 0.9019217 |
| TTK | blue | 0.4430629 | 0.8891404 |
| NEK2 | blue | 0.4415302 | 0.8622565 |
| CDC6 | blue | 0.4223794 | 0.8877682 |
| DEPDC1 | blue | 0.419818 | 0.8542489 |
| TPX2 | blue | 0.419345 | 0.9013895 |
| CDCA8 | blue | 0.4167391 | 0.9020832 |
| RAD51 | blue | 0.4112464 | 0.870926 |
| ERCC6L | blue | 0.4104088 | 0.8809704 |
| CDK1 | blue | 0.4067001 | 0.897214 |
| TRIP13 | blue | 0.4064487 | 0.8854838 |
| ASF1B | blue | 0.4063477 | 0.8600731 |
| ORC1 | blue | 0.4053694 | 0.85957 |
| MCM10 | blue | 0.4040477 | 0.8934246 |
| GINS1 | blue | 0.4014923 | 0.9100197 |
| DTL | blue | 0.3992964 | 0.8405885 |
| MCM2 | blue | 0.3986514 | 0.8610663 |
| MELK | blue | 0.3980079 | 0.8673826 |
| PLK1 | blue | 0.3951205 | 0.9116508 |
| TICRR | blue | 0.394909 | 0.9178393 |
| ASPM | blue | 0.3945547 | 0.8132015 |
| CDCA2 | blue | 0.3938097 | 0.8839586 |
| PBK | blue | 0.3895847 | 0.8128604 |
| MTFR2 | blue | 0.3893037 | 0.8542855 |
| E2F2 | blue | 0.3832449 | 0.8575272 |
| WDR62 | blue | 0.3820656 | 0.8960656 |
| KIF18B | blue | 0.3790854 | 0.9249901 |

| | | | |
|---|---|---|---|
| WDR76 | blue | 0.3753103 | 0.8045179 |
| PRR11 | blue | 0.3752499 | 0.894019 |
| BUB1 | blue | 0.3705505 | 0.8971739 |
| DLGAP5 | blue | 0.3662905 | 0.8861859 |
| CCNA2 | blue | 0.3649772 | 0.8205757 |
| KIF20A | blue | 0.3639928 | 0.8757231 |
| FANCD2 | blue | 0.3617517 | 0.9114327 |
| TOP2A | blue | 0.3608042 | 0.9035872 |
| XRCC2 | blue | 0.3594296 | 0.8871873 |
| RAD54L | blue | 0.3587781 | 0.8798465 |
| PKMYT1 | blue | 0.358288 | 0.8166639 |
| CKAP2L | blue | 0.3526731 | 0.8833491 |
| TCF19 | blue | 0.3517367 | 0.8118379 |
| PARPBP | blue | 0.3514563 | 0.8586647 |
| KIF11 | blue | 0.3502449 | 0.9110701 |
| LMNB1 | blue | 0.3501521 | 0.8827198 |
| CHAF1B | blue | 0.3481398 | 0.8701264 |
| ORC6 | blue | 0.3454413 | 0.8711912 |
| MKI67 | blue | 0.3408996 | 0.8913147 |
| PIF1 | blue | 0.3401501 | 0.8715707 |
| KIF18A | blue | 0.3374331 | 0.9021702 |
| KIF15 | blue | 0.3333868 | 0.8657131 |
| POLQ | blue | 0.3321308 | 0.8954463 |
| NCAPH | blue | 0.3315372 | 0.8974166 |
| BUB1B | blue | 0.3312897 | 0.9152923 |
| HELLS | blue | 0.3312823 | 0.8760019 |
| PRC1 | blue | 0.326967 | 0.8924303 |
| ARHGAP11A | blue | 0.3226007 | 0.8910937 |
| FOXM1 | blue | 0.3196495 | 0.8771105 |
| CENPF | blue | 0.3159993 | 0.8840696 |
| E2F8 | blue | 0.3155013 | 0.8321525 |
| CENPI | blue | 0.3122181 | 0.8745855 |
| GTSE1 | blue | 0.3009239 | 0.9165557 |
| DEPDC1B | blue | 0.2954254 | 0.8856735 |
| RAD51AP1 | blue | 0.2933366 | 0.8408958 |
| CENPE | blue | 0.2915748 | 0.8853194 |
| ANLN | blue | 0.290399 | 0.8934952 |
| KIF23 | blue | 0.2895674 | 0.9315059 |
| CENPK | blue | 0.2803071 | 0.8548599 |
| FAM72B | blue | 0.2703683 | 0.8472311 |
| UHRF1 | blue | 0.2696845 | 0.8778091 |
| CIT | blue | 0.2688118 | 0.8227112 |
| BLM | blue | 0.2655536 | 0.847759 |
| CEP55 | blue | 0.2646388 | 0.9137114 |
| RTKN2 | blue | 0.2630749 | 0.8124793 |
| GSG2 | blue | 0.2611261 | 0.8592712 |
| SHCBP1 | blue | 0.2574886 | 0.8416169 |
| KIF14 | blue | 0.2513517 | 0.8440993 |
| KIAA1524 | blue | 0.2499835 | 0.9110972 |
| KNTC1 | blue | 0.2493351 | 0.8603241 |
| STIL | blue | 0.2418659 | 0.883556 |
| CLSPN | blue | 0.2117496 | 0.8349336 |
| CTD-2510F5.4 | blue | 0.2017597 | 0.8282996 |
