# Supplementary material for: Identification and Validation of a Potential Stemness-Associated Biomarker in Hepatocellular Carcinoma
Source: Stem Cells Int. 2022 Jul 11;2022:1534593. doi: 10.1155/2022/1534593 (PMC9293570; doi:10.1155/2022/1534593)
Supplement: Supplementary Materials — Figure S1: determination of soft-thresholding power in WGCNA. (A) Analysis of the scale-free fit index and the mean connectivity for various soft-threshold powers (β = 7). (B) Histogram of connectivity distribution when β = 7. (C) Checking the scale free topology when β = 7. (D) Scatter plot of MEs in the turquoise module. Figure S2: differences in BUB1 expression and clinical characteristics in HCC: age (A), gender (B), child (C), T stage (D), M stage (E), and N stage (F). Figure S3: the mutational features in HCC. (A) The landscape of mutation in HCC. (B) The top 30 mutation genes in HCC. (C) The correlation between TP53 mutation and BUB1 expression using TIMER2.0. Supplementary Figure S4: the calibration curve for the nomogram in TCGA database (A) and ICGC database (B). Table S1: the mRNAsi score of HCC patients in the TCGA database. Table S2: the mRNAsi subtype of HCC patients in the TCGA database. Table S3: clinicopathological characteristics of HCC patients from the TCGA, ICGC, and GEO cohorts. Table S4: 737 genes in blue module by WGCNA. Table S5: 112 genes involved in PPI (MM > 0.8; GS > 0.2). Table S6: the result of MCODE method in Cytoscape. Table S7: The ‘stemness' signature in MSigDB: genes upregulated and common to 6 human embryonic stem cell lines tested. [file 1534593.f1.zip › Table S6.pdf]

Table S6. The result of MCODE method in Cytoscape.

MCODE::Clusters (1)	MCODE::N	MCODE::S	name	shared name
Cluster 0	Clustered	63.407984	BUB1	BUB1
	Unclustered	0	RTKN2	RTKN2
	Unclustered	4	WDR76	WDR76
	Unclustered	15.625731	EME1	EME1
	Unclustered	7	XRCC2	XRCC2
	Unclustered	25	E2F2	E2F2
	Unclustered	9	WDR62	WDR62
	Unclustered	36.950067	MTFR2	MTFR2
	Unclustered	45.165457	FANCD2	FANCD2
	Unclustered	46.503771	CHAF1B	CHAF1B
Cluster 0	Clustered	58.65124	UHRF1	UHRF1
Cluster 0	Clustered	56.040865	ORC1	ORC1
	Unclustered	41.702866	BLM	BLM
	Unclustered	17	PIF1	PIF1
Cluster 0	Clustered	52.551119	PKMYT1	PKMYT1
	Unclustered	35.841414	ORC6	ORC6
Cluster 0	Clustered	59.104895	CENPM	CENPM
Cluster 0	Clustered	59.43609	ASF1B	ASF1B
Cluster 0	Clustered	56.756177	E2F8	E2F8
Cluster 0	Clustered	61.074627	KIF18B	KIF18B
	Unclustered	34.963455	TCF19	TCF19
Cluster 0	Clustered	58.382294	RAD51	RAD51
	Unclustered	13	FAM72B	FAM72B
	Unclustered	32.944538	GSG2	GSG2
Cluster 0	Clustered	52.519056	CLSPN	CLSPN
	Unclustered	30.941288	KIAA1524	KIAA1524
Cluster 0	Clustered	59.025461	SPC25	SPC25
Cluster 0	Clustered	58.385417	SGOL1	SGOL1
	Unclustered	50.377578	GINS1	GINS1
	Unclustered	37.452935	PRR11	PRR11
Cluster 0	Clustered	59.28169	TROAP	TROAP
Cluster 0	Clustered	63.407984	KIF20A	KIF20A
Cluster 0	Clustered	63.407984	CEP55	CEP55
Cluster 0	Clustered	63.407984	ASPM	ASPM
Cluster 0	Clustered	63.407984	CDK1	CDK1
	Unclustered	23.794872	CIT	CIT
Cluster 0	Clustered	63.407984	TOP2A	TOP2A
Cluster 0	Clustered	63.407984	CENPF	CENPF
Cluster 0	Clustered	59.499658	KIF14	KIF14
Cluster 0	Clustered	63.407984	KIF4A	KIF4A
Cluster 0	Clustered	62.575758	PRC1	PRC1
Cluster 0	Clustered	63.407984	TPX2	TPX2
Cluster 0	Clustered	63.407984	PBK	PBK
Cluster 0	Clustered	63.407984	AURKB	AURKB
Cluster 0	Clustered	63.407984	KIF2C	KIF2C
Cluster 0	Clustered	63.407984	MELK	MELK
Cluster 0	Clustered	63.407984	CCNA2	CCNA2
Cluster 0	Clustered	63.407984	CDCA8	CDCA8
Cluster 0	Clustered	63.407984	CCNB2	CCNB2
Cluster 0	Clustered	63.604396	PLK1	PLK1
Cluster 0	Clustered	63.407984	CDC20	CDC20
Cluster 0	Clustered	63.407984	UBE2C	UBE2C
Cluster 0	Clustered	63.407984	TTK	TTK
Cluster 0	Clustered	62.619048	NEK2	NEK2
Cluster 0	Clustered	63.407984	RRM2	RRM2
Cluster 0	Clustered	63.407984	BUB1B	BUB1B
Cluster 0	Clustered	63.407984	KIF15	KIF15
Cluster 0	Clustered	63.407984	NUF2	NUF2
Cluster 0	Clustered	63.407984	BIRC5	BIRC5

Cluster 0	Clustered	61.768968	SHCBP1	SHCBP1
Cluster 0	Clustered	62.532468	CENPA	CENPA
Cluster 0	Clustered	63.407984	EXO1	EXO1
Cluster 0	Clustered	63.604396	HJURP	HJURP
Cluster 0	Clustered	61.203999	CDKN3	CDKN3
Cluster 0	Clustered	59.715789	DEPDC1	DEPDC1
Cluster 0	Clustered	63.985263	FOXM1	FOXM1
Cluster 0	Clustered	63.407984	MKI67	MKI67
Cluster 0	Clustered	62.016577	DTL	DTL
Cluster 0	Clustered	54.707692	CENPK	CENPK
Cluster 0	Clustered	58.968792	CKAP2L	CKAP2L
Cluster 0	Clustered	63.985263	ZWINT	ZWINT
Cluster 0	Clustered	63.407984	CDC45	CDC45
Cluster 0	Seed	63.985263	CDCA2	CDCA2
Cluster 0	Clustered	63.407984	CDCA5	CDCA5
Cluster 0	Clustered	58.900256	ARHGAP11	ARHGAP11A
Cluster 0	Clustered	61.725222	KIAA0101	KIAA0101
Cluster 0	Clustered	61.844211	KNTC1	KNTC1
Cluster 0	Clustered	57.601405	HELLS	HELLS
Cluster 0	Clustered	62.822967	SKA3	SKA3
Cluster 0	Clustered	61.506494	RAD54L	RAD54L
Cluster 0	Clustered	58.735733	ERCC6L	ERCC6L
Cluster 0	Clustered	63.81203	MCM10	MCM10
Cluster 0	Clustered	61.852174	KIFC1	KIFC1
Cluster 0	Clustered	54	STIL	STIL
Cluster 0	Clustered	59.003805	SKA1	SKA1
Cluster 0	Clustered	52.044262	PARPBP	PARPBP
Cluster 0	Clustered	60.520344	CDCA3	CDCA3
Cluster 0	Clustered	60.701053	GTSE1	GTSE1
Cluster 0	Clustered	52.551119	TICRR	TICRR
Cluster 0	Clustered	57.191346	CDC25A	CDC25A
Cluster 0	Clustered	60.969309	CDT1	CDT1
Cluster 0	Clustered	55.111111	CENPI	CENPI
Cluster 0	Clustered	63.407984	CENPE	CENPE
Cluster 0	Clustered	59.982441	MCM2	MCM2
Cluster 0	Clustered	59	DEPDC1B	DEPDC1B
Cluster 0	Clustered	52.91486	POLQ	POLQ
Cluster 0	Clustered	59.096651	KIF18A	KIF18A
Cluster 0	Clustered	63.407984	NDC80	NDC80
	Unclustered	49.84917	LMNB1	LMNB1
Cluster 0	Clustered	63.407984	KIF11	KIF11
Cluster 0	Clustered	63.81203	KIF23	KIF23
Cluster 0	Clustered	63.407984	CCNB1	CCNB1
Cluster 0	Clustered	63.407984	NCAPG	NCAPG
Cluster 0	Clustered	63.407984	DLGAP5	DLGAP5
Cluster 0	Clustered	63.407984	NCAPH	NCAPH
Cluster 0	Clustered	61.195716	RAD51AP1	RAD51AP1
Cluster 0	Clustered	61.656338	OIP5	OIP5
Cluster 0	Clustered	59.355332	MYBL2	MYBL2
Cluster 0	Clustered	62.320675	CDC6	CDC6
Cluster 0	Clustered	62.532468	TRIP13	TRIP13
Cluster 0	Clustered	60.652716	ANLN	ANLN
