# Supplementary material for: Identification and Validation of a Potential Stemness-Associated Biomarker in Hepatocellular Carcinoma
Source: Stem Cells Int. 2022 Jul 11;2022:1534593. doi: 10.1155/2022/1534593 (PMC9293570; doi:10.1155/2022/1534593)
Supplement: Supplementary Materials — Figure S1: determination of soft-thresholding power in WGCNA. (A) Analysis of the scale-free fit index and the mean connectivity for various soft-threshold powers (β = 7). (B) Histogram of connectivity distribution when β = 7. (C) Checking the scale free topology when β = 7. (D) Scatter plot of MEs in the turquoise module. Figure S2: differences in BUB1 expression and clinical characteristics in HCC: age (A), gender (B), child (C), T stage (D), M stage (E), and N stage (F). Figure S3: the mutational features in HCC. (A) The landscape of mutation in HCC. (B) The top 30 mutation genes in HCC. (C) The correlation between TP53 mutation and BUB1 expression using TIMER2.0. Supplementary Figure S4: the calibration curve for the nomogram in TCGA database (A) and ICGC database (B). Table S1: the mRNAsi score of HCC patients in the TCGA database. Table S2: the mRNAsi subtype of HCC patients in the TCGA database. Table S3: clinicopathological characteristics of HCC patients from the TCGA, ICGC, and GEO cohorts. Table S4: 737 genes in blue module by WGCNA. Table S5: 112 genes involved in PPI (MM > 0.8; GS > 0.2). Table S6: the result of MCODE method in Cytoscape. Table S7: The ‘stemness' signature in MSigDB: genes upregulated and common to 6 human embryonic stem cell lines tested. [file 1534593.f1.zip › Table S7.pdf]

Table S7. The 'stemness' signature in MSigDB: genes up-regulated and common to 6 human embryonic stem cell lir
ARL5B
AURKB
BRIX1
CACHD1
CALU
CCNB1
CCNC
CCT8
CDK1
CRABP1
CYP26A1
DDX21
DSG2
DTL
EIF4A1
ELOVL6
EPRS1
FABP5
FAM83D
GAL
GDF3
GJA1
GNL3
GSX1
HDAC2
HMGA1
HMGB2
HNRNPAB
HSPA4
IDH1
IFITM1
IGF2BP2
IMPDH2
JADE1
KIF4A
KPNA2
KRT8
LDHB
LIN28A
LRRN1
MAD2L2
MGST1
MTHFD1
MTHFD2
NANOG
NASP
NME2
NPM1
OR7E33P
PITX2
PODXL
POU5F1
PSIP1
PSMA2
PSMA3
PTTG1
RCC2
RPL24
RPL4
RPL6

RPL7
RPLP0
RPS24
RPSAP44
RSL24D1
SEMA6A
SEPHS1
SERPINH1
SET
SFRP2
SLC16A1
SMS
SNRPF
SRSF7
SSB
TDGF1
TK1
TNNC2
TNNT1
TPX2
TUBB
TUBB6
ZNF257
ZNF43

ıes tested.
